# Metabolic syndrome-related prognostic index: Predicting biochemical recurrence and differentiating between cold and hot tumors in prostate cancer

**DOI:** 10.3389/fendo.2023.1148117

**Published:** 2023-03-24

**Authors:** Congzhe Ren, Qihua Wang, Shangren Wang, Hang Zhou, Mingming Xu, Hu Li, Yuezheng Li, Xiangyu Chen, Xiaoqiang Liu

**Affiliations:** ^1^ Department of Urology, Tianjin Medical University General Hospital, Tianjin, China; ^2^ Department of Urology, Shanxian Central Hospital (Affiliated Huxi Hospital of Jining Medical University), Heze, China

**Keywords:** metabolic syndrome, prostate cancer, biochemical recurrence, prognostic model, immune microenvironment

## Abstract

**Background:**

The prostate, as an endocrine and reproductive organ, undergoes complex hormonal and metabolic changes. Recent studies have shown a potential relationship between metabolic syndrome and the progression and recurrence of prostate cancer (PCa). This study aimed to construct a metabolic syndrome-related prognostic index (MSRPI) to predict biochemical recurrence-free survival (BFS) in patients with PCa and to identify cold and hot tumors to improve individualized treatment for patients with PCa.

**Methods:**

The Cancer Genome Atlas database provided training and test data, and the Gene Expression Omnibus database provided validation data. We extracted prognostic differentially expressed metabolic syndrome-related genes (DEMSRGs) related to BFS using univariate Cox analysis and identified potential tumor subtypes by consensus clustering. The least absolute shrinkage and selection operator (LASSO) algorithm and multivariate Cox regression were used to construct the MSRPI. We further validated the predictive power of the MSRPI using KaplanMeier survival analysis and receiver operating characteristic (ROC) curves, both internally and externally. Drug sensitivity was predicted using the half-maximal inhibitory concentration (IC50). Finally, we explored the landscape of somatic mutations in the risk groups.

**Results:**

Forty-six prognostic DEMSRGs and two metabolic syndrome-associated molecular clusters were identified. Cluster 2 was more immunogenic. Seven metabolic syndrome-related genes (*CSF3R*, *TMEM132A*, *STAB1*, *VIM*, *DUOXA1*, *PILRB*, and *SLC2A4*) were used to construct risk equations. The high-risk index was significantly associated with a poor BFS, which was also validated in the validation cohort. The area under the ROC curve (AUC) for BFS at 1-, 3-, and 5- year in the entire cohort was 0.819, 0.785, and 0.772, respectively, demonstrating the excellent predictive power of the MSRPI. Additionally, the MSRPI was found to be an independent prognostic factor for BFS in PCa. More importantly, MSRPI helped differentiate between cold and hot tumors. Hot tumors were associated with the high-risk group. Multiple drugs demonstrated significantly lower IC50 values in the high-risk group, offering the prospect of precision therapy for patients with PCa.

**Conclusion:**

The MSRPI developed in this study was able to predict biochemical recurrence in patients with PCa and identify cold and hot tumors. MSRPI has the potential to improve personalized precision treatment.

## 1 Introduction

Prostate cancer (PCa) is the second most common cancer in men worldwide (1). There were more than 1,400,000 new cases of PCa worldwide and more than 370,000 deaths as a result in 2020 (2). The incidence and mortality of PCa is positively correlated with age, with a mean age at diagnosis of 66 years (3). In addition, there are significant geographic differences in the incidence and mortality of PCa, which is particularly common in developed countries. In the United States, PCa is the leading cause of cancer events and the second most common cause of cancer death in men (4). In comparison, Asia has the lowest incidence and mortality rates. However, with economic development and westernization of lifestyle, the incidence of PCa is rapidly increasing (5).

Radical prostatectomy (RP) is the primary treatment option for localized PCa. Nevertheless, owing to persistently elevated prostate-specific antigen (PSA) levels, nearly 50% of patients still experience biochemical recurrence (BCR) after surgery, suggesting a proclivity for poor prognosis (6). Nonetheless, it is not the case that every patient undergoing BCR will suffer from progressive disease (7). The impact of BCR on survival was mainly observed in patients with specific clinical risk factors such as a high Gleason score after RP (8). Long-term follow-up of patients with PCa often spans decades, and many patients do not die from PCa, making it difficult to reach the endpoint of overall survival (OS) in clinical trials. Thus, BCR serves as an intermediate clinical endpoint that can indicate clinical progression when the disease is at a low load (9, 10). Currently, there are no accepted molecular clusters and personalized scoring criteria associated with BCR, despite efforts to identify biomarkers or subtypes of PCa for BCR prediction (11).

The metabolic syndrome (MetS) is a set of combined clinical risk factors, primarily including obesity, insulin resistance, dyslipidemia and hypertension, and is significantly associated with an increased risk of type 2 diabetes and cardiovascular disease. The WHO definition for MetS includes insulin resistance as an essential component. However, the National Cholesterol Education Program Adult Treatment Panel III (NCEP ATP III) does not require that criterion. In contrast, the NCEP ATP III considers patients who meet three of the five criteria of obesity, hyperlipidemia, hypertension, elevated blood glucose levels and reduced HDL cholesterol levels to be diagnosed with MetS, which is considered by clinicians to be more applicable to clinical practice (12). The prevalence of MetS is highly correlated with age. In France, the prevalence is 5.6% and 17.5% in people aged 30-39 years and 60-64 years, respectively. Furthermore, in the US population, the prevalence rises from 7% in participants aged 20–29 years to 44% in those aged 60–69 years (13). From 2015 to 2017, the prevalence of MetS among Chinese residents aged 20 years and older was 31.1%. In addition, due to the unique urban-rural differences in China, the prevalence of MetS is relatively higher in urban areas than in rural areas (14). In recent years, the prevalence of MetS has increased significantly with the increase in obesity rates among adolescents (13). This particular syndrome is the result of the interaction between multiple genes and the environment. Similarly, cancer, as a group of multifactorial diseases, is thought to be correlated with genetic and metabolic abnormalities (15). Consistent with the original theory of Otto Warburg, there is growing evidence that cancer is principally a metabolic disease (16). In the past few years, the association of MetS with cancer has been documented, including PCa, but the exact mechanism underlying the relationship remains unclear (17, 18). According to studies published to date, obesity and insulin resistance (IR), the central clinical features of MetS, are associated with a high risk of cancer at multiple sites (18). Generally, obesity can promote IR (19), subsequently leading to hyperinsulinemia and hyperglycemia. Hyperinsulinemia contributes to cell mitosis. Overproduction of reactive oxygen species (ROS) by hyperglycemia causes oncogenic mutations and carcinogenesis. In addition, hyperglycemia decreases sex hormone binding globulin, leading to elevated insulin-like growth factor-1 (IGF-1) and suppressed apoptosis. Overall, this process leads to tumorigenesis (20).

As a male-specific endocrine and reproductive organ, the prostate harbors complex hormonal and metabolic variations. Therefore, MetS and PCa may be linked intrinsically (21). It has been investigated that the presence of MetS is correlated with malignant outcomes of PCa, especially BCR (22). An et al. reported in a real-world study that metastatic prostate cancer (mPCa) patients with MetS traits were more likely to progress to castration-resistant prostate cancer (CRPC) and had lower PSA remission rates and shorter survival times (23). However, to the best of our knowledge, studies addressing MetS and BCR in PCa are limited.

In this era of immune-targeted therapies, the concept of hot and cold tumors has been proposed, laying the foundation for precise patient stratification and individualized treatment (24). PCa has been portrayed as an immunological desert, and the majority of PCa patients respond weakly to checkpoint inhibitors, such as anti-PD1 or anti-CTLA-4 (25). Therefore, our purpose was to identify prognostic metabolic syndrome-related genes associated with BCR in PCa. These genes may be involved in the metabolic process of PCa progression and may be potential targets for controlling disease progression and recurrence. We then constructed a metabolic syndrome-related prognostic index (MSRPI) using seven genes to stratify patients and effectively identify hot tumors to provide a basis for establishing individualized treatment regimens and drug choices.

## 2 Materials and methods

### 2.1 Data collection and processing

We downloaded the transcriptomic and clinical data of PCa patients from The Cancer Genome Atlas (TCGA) (https://portal.gdc.cancer.gov/) (26). In this study, we combined the data types of “biochemical recurrence” and “new tumor event after initial treatment” to define the state of BCR. In addition, “days to first biochemical recurrence” or “days to new tumor event after initial treatment” was identified as the time to BCR. For the samples with the data of “biochemical recurrence” and “days to first biochemical recurrence”, we considered them as the state of BCR and the time to BCR, respectively. In those with data of “biochemical recurrence” but no “days to first biochemical recurrence,” we consider “new tumor event after initial treatment” and “days to new tumor event after initial treatment” as the state of BCR and the time to BCR, respectively; in those with data of “days to first biochemical recurrence” but no “biochemical recurrence,” we utilized “new tumor event after initial treatment” as the state of BCR. BCR-free survival (BFS) was defined as the interval from radical treatment to the first BCR or death. Finally, we screened 400 PCa samples with the state of BCR and the time to BCR, which were randomly divided into training (n = 200) and test cohorts (n = 200). The validation cohort was the dataset GSE70769 extracted from the Gene Expression Omnibus (GEO) database (http://www.ncbi.nlm.nih.gov/geo/).

Differentially expressed genes (DEGs) for PCa were obtained from Gene Expression Profiling Interactive Analysis (GEPIA) (http://gepia2.cancer-pku.cn/) using the Limma differential method (|log2FC| >1, padj <0.01) (**Supplementary Data sheet 1**) (27). The metabolic syndrome network containing 1,243 genes was acquired from the Molecular Signatures Database (MsigDB) (http://www.broad.mit.edu/gsea/msigdb/) (**Supplementary Data sheet 2**) (28, 29). We screened for differentially expressed metabolic syndrome-related genes (DEMSRGs) associated with BFS in the training cohort using univariate Cox regression analysis.

### 2.2 Functional enrichment

By uploading 208 DEMSRGs into the Database for Annotation, Visualization, and Integrated Discovery (DAVID) (30), we obtained the results of Gene Ontology (GO) analysis. The first ten results are displayed in ascending order of p-values. Gene set enrichment analysis (GSEA) was used for Kyoto Encyclopedia of Genes and Genomes (KEGG) pathways using GSEA software (28). P < 0.05 was considered statistically significant.

### 2.3 Clusters based on 46 prognostic DEMSRGs

Based on the prognostic DEMSRGs, potential molecular clusters were explored using the ConsensusClusterPlus R package (31). Principal component analysis (PCA) was performed using the Rtsne R package.

### 2.4 A novel metabolic syndrome-related prognostic index for BCR-free survival

We performed least absolute shrinkage and selection operator (LASSO) on the 46 prognostic DEMSRGs and extracted 13 DEMSRGs at the appearance of minimal partial likelihood deviance. Seven of these 13 DEMSRGs were subsequently screened using multivariate Cox regression analysis to participate in the construction of the MSRPI.

The MSRPI was calculated using the following formula:


MSRPI=gene (A) expression*coef (A)+gene (B) expression*coef (B)+…+gene (i) expression*coef (i).

Patients in the training cohort, test cohort, and entire cohort were divided into high- and low-risk groups based on the median MSRPI of the training cohort. KaplanMeier survival analysis was performed using the survival and survminer R packages. P < 0.05 was considered statistically significant. Time-dependent receiver operating characteristic (ROC) curves were generated using the timeROC R package to verify the predictive power of different factors on BFS.

### 2.5 A predictive nomogram and calibration

The patient data for constructing the nomogram were derived from the entire TCGA cohort. Using the rms R package, the nomogram was constructed using age, Gleason score, T stage, N stage and risk to predict 1-, 3-, and 5-year BFS. The calibration curve demonstrates the accuracy of the prediction.

### 2.6 Immune infiltrating cells and activation of immune checkpoints

Using the gsva R package, single-sample gene set enrichment analysis (ssGSEA) was used to calculate the scores of immune-infiltrating cells and immune function. Using the “infiltration estimation for tcga” file from the TIMER2.0 (http://timer.cistrome.org/) (32), relying on limma, scales, ggplot2, ggtext and pheatmap R packages, the immune infiltration analysis was performed using different methods and the results were visualized using a bubble chart and a heat map. In addition, we compared the tumor microenvironment (TME) scores (including stromal score, immune score, and estimate score) by estimating the R package and the activation of immune checkpoints using the ggpubr R package between different subgroups.

### 2.7 Drug sensitivity and landscape of somatic mutation

To evaluate the treatment response, we used the pRRophetic R package to calculate half-maximal inhibitory concentrations (IC50) for each patient using the Genomics of Drug Sensitivity in Cancer (GDSC) (https://www.cancerrxgene.org/) (33). Finally, we explored the landscape of somatic mutations and calculated the tumor mutational burden (TMB) of patients in the risk groups using the maftools R package. Single-nucleotide mutation data of the patients were obtained from TCGA database.

## 3 Results

### 3.1 Functional enrichment and cox regression analysis

**Figure 1:**
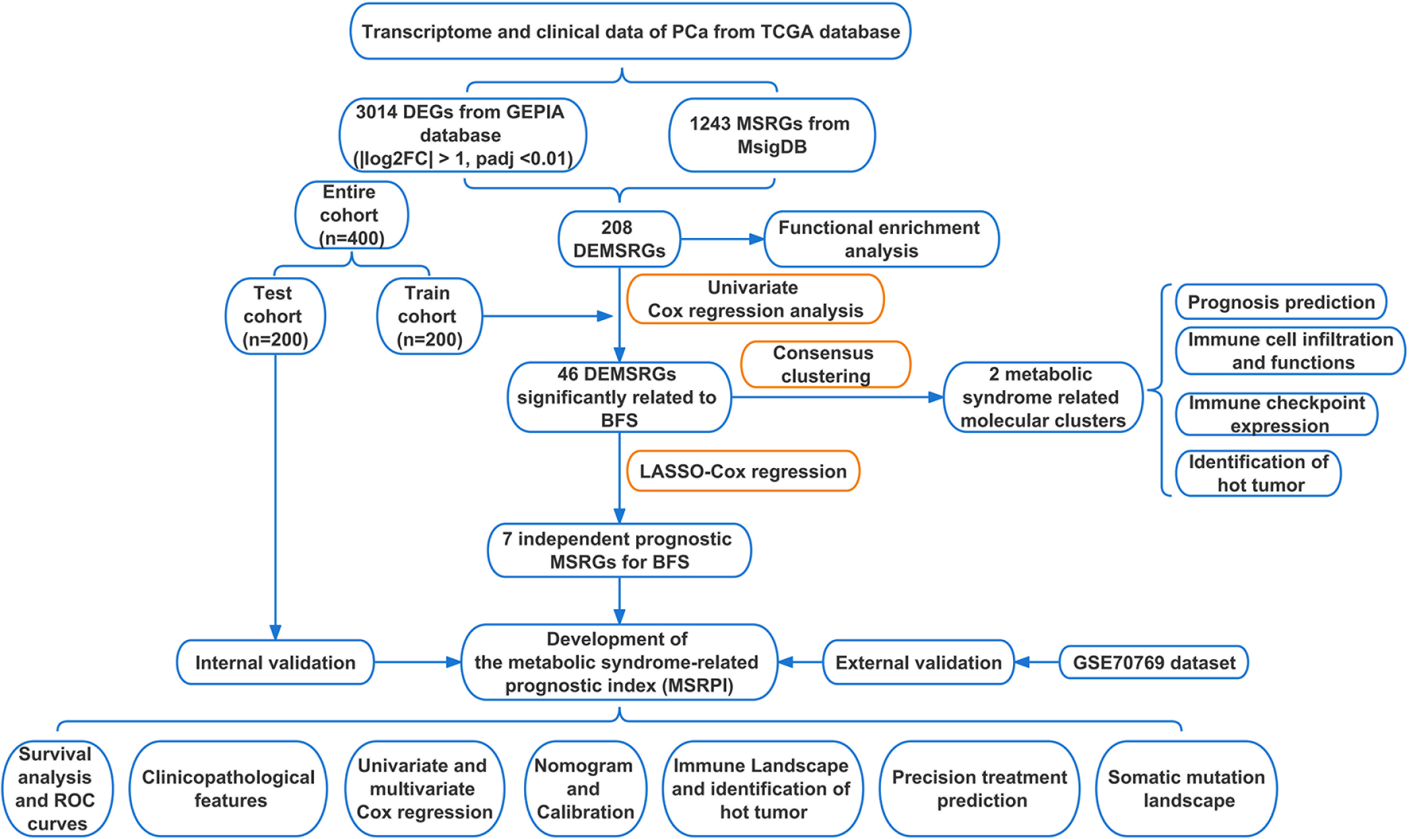
Flow chart of this study. TCGA, The Cancer Genome Atlas; DEGs, differentially expressed genes; GEPIA, Gene Expression Profiling Interactive Analysis; MSRGs, metabolic syndrome-related genes; MsigDB, molecular signatures database; DEMSRGs, differentially expressed metabolic syndrome-related genes; BFS, BCR-free survival; LASSO, least absolute shrinkage and selection operator; ROC, receiver operating characteristics.

**Figure 2:**
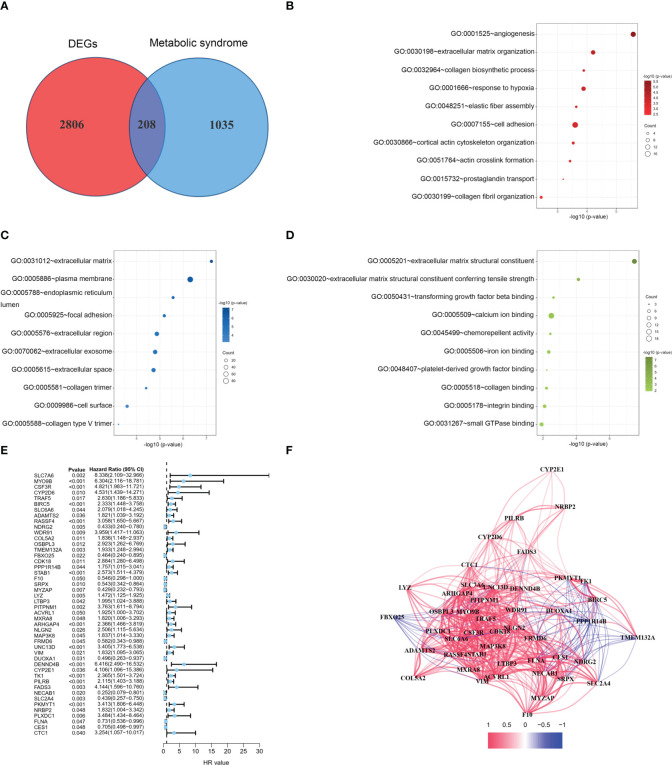
GO analyses and identification of prognostic DEMSRGs. **(A)** Venn diagram of DEGs and MSRGs. **(B–D)** BP, CC and MF of 208 DEMSRGs. **(E)** Univariable Cox regression to identify 46 DEMSRGs related to BFS in the train cohort. **(F)** Correlation network of 46 DEMSRGs.

The flow of this study is presented in **Figure 1**. The clinical characteristics of the training cohort and the testing cohort are showed in **Table 1**. We identified 208 DEMSRGs (**Figure 2A**) and the results of their functional enrichment analysis are presented in **Figures 2B–D**. Based on the results of GO enrichment analysis, the most relevant biological processes (BP) were angiogenesis, extracellular matrix organization, and collagen biosynthetic process (**Figure 2B**), and the most relevant cellular components (CC) were the extracellular matrix, plasma membrane, and endoplasmic reticulum lumen (**Figure 2C**). The most relevant molecular functions (MF) were extracellular matrix structural constituent, extracellular matrix structural constituent conferring tensile strength, and transforming growth factor beta binding (**Figure 2D**). Subsequently, 46 prognostic DEMSRGs related to BFS were screened using univariate Cox regression analysis (P < 0.05) (**Figure 2E**). The correlation network of the 46 DEMSRGs is shown in **Figure 2F**.

**Table 1:** The clinical characteristics of PCa patients in the training and testing cohorts.

Characteristics	Training cohort	Testing cohort
**Sample**	200	200
**Age**	–	–
<=60	90	94
>60	110	106
**Gender**	–	–
Male	200	200
**T stage**	–	–
T2a	3	5
T2b	8	1
T2c	52	82
T3a	72	55
T3b	55	55
T4	5	2
Unknown	5	0
**N stage**	–	–
N0	144	142
N1	32	28
Unknown	24	30
**M stage**	–	–
M0	190	185
M1	0	2
Unknown	10	13
**Gleason score**	–	–
GS=6	17	21
GS=7	103	95
GS=8	28	22
GS=9	50	61
GS=10	2	1

### 3.2 Identification of two metabolic syndrome-associated molecular clusters

**Figure 3:**
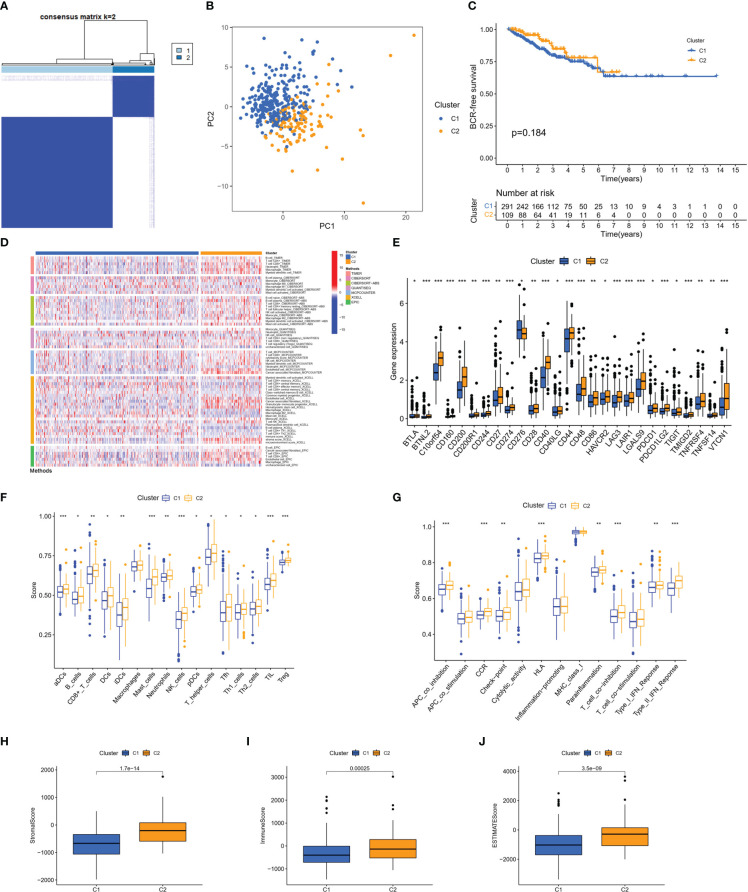
Identification of two metabolic syndrome-associated clusters. **(A)** Consensus clustering. **(B)** PCA. **(C)** KM survival analysis of clusters. **(D)** Immune infiltration analysis of the different platforms. **(E)** Expression of immune checkpoints. **(F, G)** SsGSEA of immune infiltrating cells and immune function. **(H–J)** Stromal score, immune score, and ESTIMATE score for two clusters. *p<0.05, **p<0.01, ***p<0.001.

Based on these 46 genes, consensus clustering analysis was performed to classify patients into two clusters (**Figure 3A**). We then performed a principal component analysis (PCA) to clearly distinguish the two clusters (**Figure 3B**). There was no significant difference in the BFS between the two clusters (**Figure 3C**). However, according to the analyses of the different platforms, cluster 2 was infiltrated more by immune cells (**Figure 3D**). In addition, immune checkpoints, such as CD274, CD40, CD44, and LAG-3, showed a greater degree of activation in cluster 2 (**Figure 3E**). Single-sample GSEA (ssGSEA) was used to score immune cell infiltration and function. The results showed that cluster 2 had a higher percentage of infiltrating B cells, CD8+ T cells, dendritic cells (DCs), T helper cells, and regulatory T (Treg) cells than cluster 1 (**Figure 3F**). The results also revealed that cluster 2 had a higher score for a series of immune functions, such as parainflammation, T-cell co-stimulation, and type II IFN response (**Figure 3G**). Consistently, the stromal, immune, and Estimation of STromal and Immune cells in MAlignant Tumor tissues using Expression data (ESTIMATE) scores in cluster 2 were markedly higher than those in cluster 1 (**Figures 3H–J**). Additionally, we further analyzed the correlation coefficients of each DEMSRGs with immune cell infiltration in the two clusters (**Supplementary Figure 1**). Overall, cluster 2 was more immunogenic, indicating that cluster 2 was hotter than cluster 1.

### 3.3 Construction and validation of MSRPI

**Figure 4:**
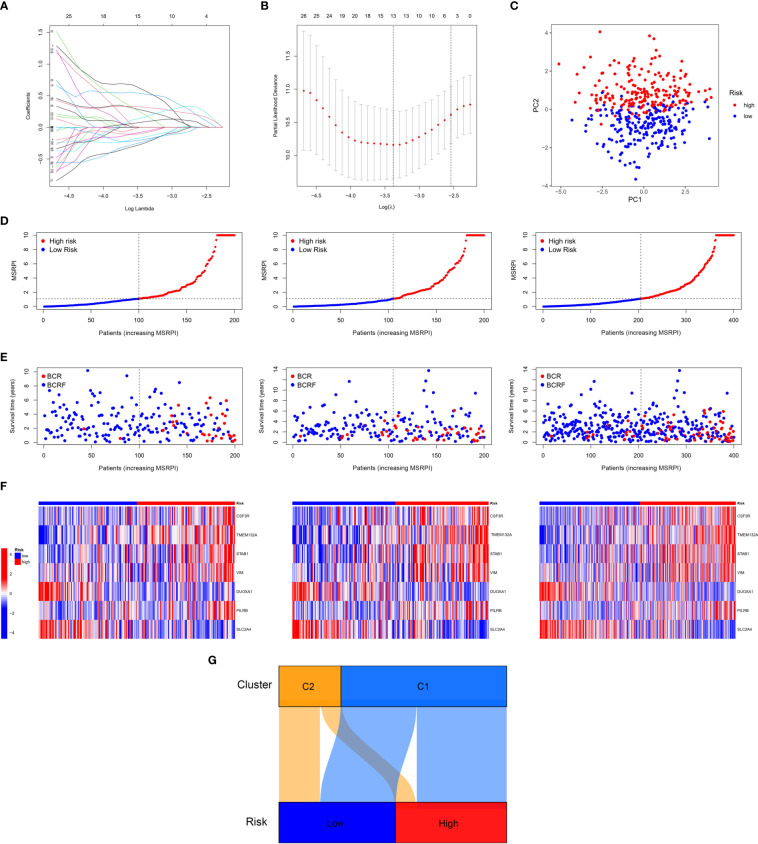
Construction of the MSRPI. **(A, B)** LASSO regression analysis **(C)** and PCA. The distribution of **(D)** MSRPI, **(E)** survival status and **(F)** gene expression of the train, test, and entire TCGA cohort. **(G)** The Sankey diagram of clusters and risk groups.

First, we performed LASSO regression on the 46 DEMSRGs in the training cohort, extracting 13 DEMSRGs (**Figures 4A, B**). Subsequently, multivariate Cox regression was performed on these 13 DEMSRGs, seven of which were screened for the construction of the MSRPI (including *CSF3R*, *TMEM132A*, *STAB1*, *VIM*, *DUOXA1*, *PILRB*, and *SLC2A4*).

We calculated MSRPI with the formula:


$$\begin{array}{l} \text{MSRPI}=(1.405977046)*CSF3R+(0.69687348)*TMEM132A+(0.898243278)*\\ STAB1+(0.594172062)*VIM+(-1.073071594)*DUOXA1+(0.508225585)*\\ PILRB+(-0.956336578)*SLC2A4. \end{array}$$

The MSRPI was calculated for each patient in the training, test, entire cohort, and validation cohorts. As shown in **Figure 4C**, the principal component analysis (PCA) was able to distinguish high-risk samples from low-risk samples. Patients in the training, test, and entire cohorts were divided into high- and low-risk groups (**Figure 4D**). The BFS status and expression heat map of the seven DEMSRGs of the training, testing, and entire cohort are shown in **Figures 4E, F**, respectively. The Sankey diagram presented the relationship between the clusters and risk groups (**Figure 4G**).

**Figure 5:**
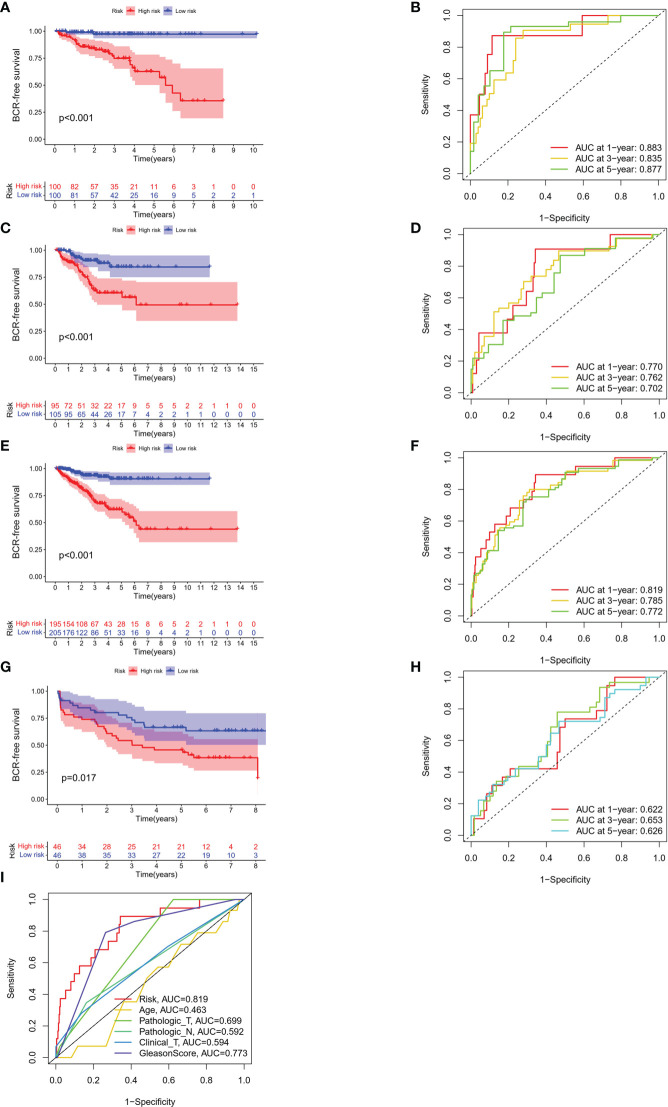
Survival analyses of different cohorts. KM analyses of BFS and 1-, 3- and 5-year ROC curves in the **(A, B)** train, **(C, D)** test, **(E, F)** entire and **(G, H)** validation cohort. **(I)** The ROC curve of risk and other clinical factors.

**Figure 6:**
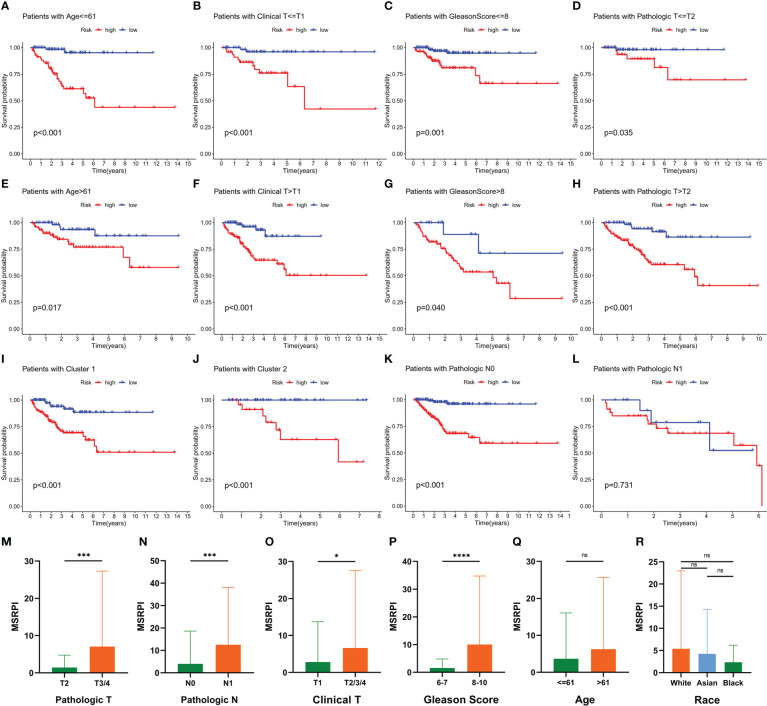
Prognostic value of MSRPI and its relationship with clinicopathological factors. KM curves of BFS in patients stratified by **(A, E)** age, **(B, F)** clinical T, **(C, G)** Gleason score, **(D, H)** pathologic T, **(K, L)** pathologic N, **(I, J)** and cluster. Relationships between MSRPI and other factors including **(M)** pathologic T, **(N)** pathologic N, **(O)** clinical T, **(P)** Gleason score, **(Q)** age and **(R)** race. *p < 0.05, **p < 0.01, ***p < 0.001, ****p < 0.0001 and “ns” means not statistically significant.

The BFS of high-risk patients in the training (**Figure 5A**), test (**Figure 5C**), entire (**Figure 5E**), and validation cohorts (**Figure 5G**) was significantly lower than that of the low-risk patients. We utilized ROC curves to evaluate the sensitivity and specificity of the MSRPI for biochemical recurrence. The area under the ROC curve (AUC) represents the outcome of the ROC. The 1-, 3-, and 5-year AUC of the training cohort were 0.883, 0.835, and 0.877, respectively (**Figure 5B**). Those of the test cohort were 0.770, 0.762, and 0.702 (**Figure 5D**), respectively. The 1-, 3-, and 5-year AUC for the entire cohort were 0.819, 0.785, and 0.772 (**Figure 5F**), and those of the validation cohort were 0.622, 0.653, and 0.626, respectively (**Figure 5H**). The risk level based on the MSRPI showed remarkable predictive power compared to clinical factors (**Figure 5I**). BFS remained lower in the high-risk group in the subgroups of age (**Figures 6A, E**), clinical T (**Figures 6B, F**), Gleason score (**Figures 6C, G**), pathologic T (**Figures 6D, H**), clusters (**Figures 6I, J**), and pathologic N0 (**Figure 6K**). In the pathological N1 subgroup, the difference in BFS between the risk groups was not statistically significant (**Figure 6L**). Moreover, we found a significant positive correlation between the MSRPI and several staging indicators (pathologic T, pathologic N, clinical T, and Gleason score) of PCa (P < 0.05) (**Figures 6M–P**). In contrast, the MSRPI did not show a significant correlation with age (**Figure 6Q**) or race (**Figure 6R**).

### 3.4 Construction of a nomogram predicting the 1-, 3- and 5-year BFS

**Figure 7:**
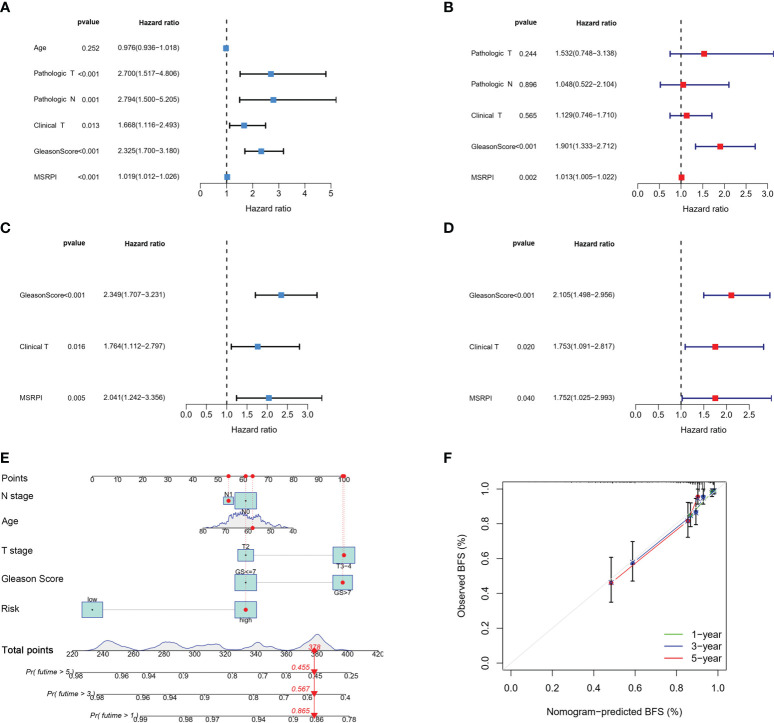
Cox regression analysis and construction of the nomogram. Univariate and multivariate Cox regression of MSRPI and other clinical parameters in the **(A, B)** entire TCGA and **(C, D)** validation cohorts. **(E)** Nomogram based on T stage, N stage, age, Gleason score and risk level. **(F)** Calibration plot of nomogram.

We performed univariate and multivariate Cox regression analyses on the MSRPI and clinical factors in TCGA (**Figures 7A, B**) and validation cohorts (**Figures 7C, D**), respectively. The MSRPI was an independent predictor of BFS in PCa patients in both cohorts (TCGA: univariate: HR = 1.019, P < 0.001; multivariate: HR = 1.013, P = 0.002; GSE70769: univariate: HR = 2.041, P = 0.005; multivariate: HR = 1.752, P = 0.040). In addition, we found that Gleason score was also an independent prognostic parameter (TCGA: univariate: HR = 2.325, P < 0.001; multivariate: HR = 1.901, P < 0.001; GSE70769: univariate: HR = 2.349, P < 0.001; multivariate: HR = 2.105, P < 0.001) (**Figures 7A–D**).

According to age, Gleason score, T stage, N stage and risk level based on the MSRPI, we constructed a nomogram to predict the 1-, 3-, and 5-year BFS of patients with PCa (**Figure 7E**). The calibration plot showed the excellent predictive power of the nomogram for 1-, 3-, and 5-year BFS (**Figure 7F**).

### 3.5 Differentiating between cold and hot tumors and precision treatment in risk groups

**Figure 8:**
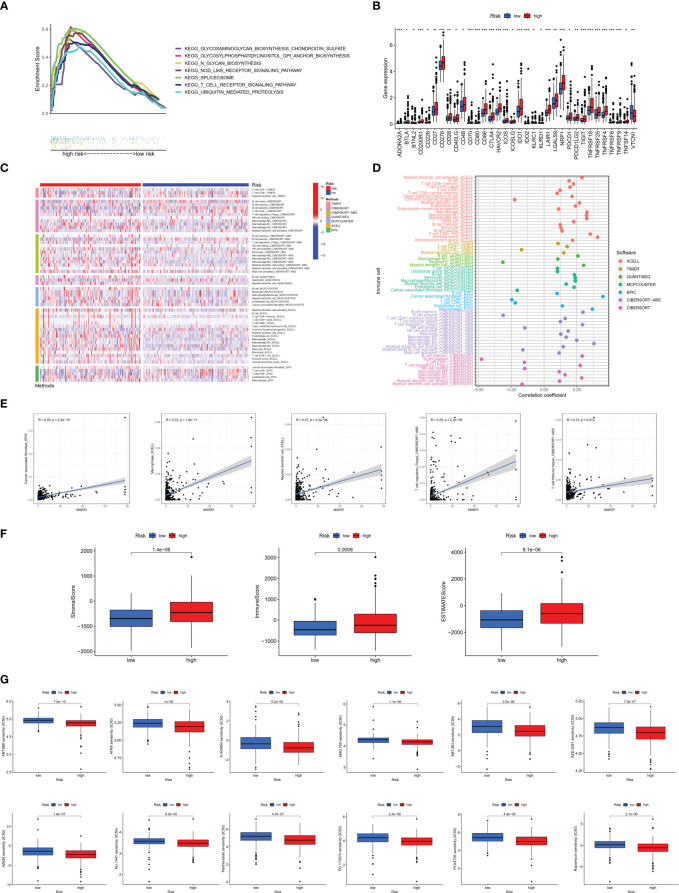
Immune infiltration analysis between the high- and low-risk groups. **(A)** Main results of GSEA in high-risk group. **(B)** Immune checkpoints activation. **(C)** Heat map of immune infiltration. **(D, E)** Association of infiltrating immune cells with MSRPI based on different software platforms. **(F)** Stromal score, immune score, and ESTIMATE score for two risk groups. **(G)** Drug sensitivity prediction in risk groups. *p<0.05, **p<0.01, ***p<0.001.

GSEA was conducted for KEGG functional enrichment analysis in the high-risk group. Glycosaminoglycan biosynthesis, chondroitin sulfate, glycosylphosphatidylinositol (GPI) anchor biosynthesis, N-glycan biosynthesis, nod-like receptor signaling pathway, spliceosome, T cell receptor signaling pathway, and ubiquitin-mediated proteolysis were the main KEGG enriched pathways in the high-risk group (**Figure 8A**). In addition, we found that almost all immune checkpoints were highly activated in the high-risk group, including CD276, CTLA4, HAVCR2 (TIM3), and NRP1 (**Figure 8B**). In addition, the landscape of immune infiltration based on different platforms demonstrated that the high-risk group possessed a richer immune abundance (**Figure 8C**). We further explored the correlation between the MSRPI and immune cell infiltration. According to the results of the analysis employing different software platforms, more immune cells were significantly and positively associated with the MSRPI (**Figure 8D**), such as cancer-associated fibroblasts in EPIC, macrophages and myeloid dendritic cells in XCELL, and Tregs and T cell follicular helper in CIBERSORT-ABS (**Figure 8E**). The stromal, immune, and ESTIMATE scores were also higher in the high-risk group (**Figure 8F**). In summary, these indicate that the high-risk group has immunologically hot tumors and are more likely to benefit from immunotherapy. We screened 34 targeted agents that showed lower IC50 values in the high-risk group in the treatment of PCa, 12 of which were displayed, such as AMG706 (motesanib) and rapamycin (**Figure 8G**).

### 3.6 Landscape of somatic mutation in risk groups

**Figure 9:**
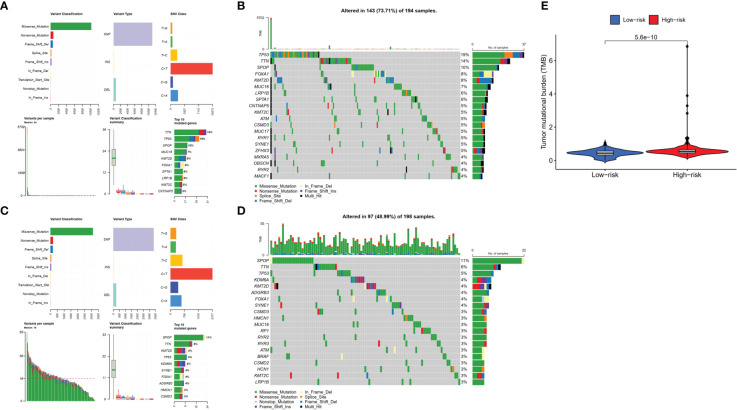
Tumor mutations diagram in risk groups. Mutational landscape in the **(A, B)** high- and **(C, D)** low-risk groups. **(E)** TMB in the high- and low-risk groups.

To explore the difference in somatic mutations in the risk groups, we calculated the proportion and type of mutation for each patient and the tumor mutational burden (TMB) of both groups. **Figures 9A–D** shows the landscape of mutations in the high- and low-risk groups, respectively. The top five genes with the highest mutation rates in the high-risk group were *TP53* (19%), *TTN* (14%), *SPOP* (10%), *FOXA1* (8%), and *KMT2D* (8%). *SPOP* (11%), *TTN* (6%), *TP53* (5%), *KDM6A* (4%), and *KMT2D* (4%) showed the highest mutation rates in the low-risk group. TP53 and TTN had a higher proportion of mutations in the high-risk group, whereas SPOP had a higher proportion of mutations in the low-risk group. Moreover, TMB was higher in the high-risk group than in the low-risk group (**Figure 9E**).

## 4 Discussion

Despite tremendous progress in recent years in oncologic therapeutic strategies targeting immune checkpoints, therapeutic efficacy is not satisfactory in a wide range of PCa patients owing to the immunosuppressive tumor microenvironment of PCa. Therefore, we introduce the concept of hot and cold tumors, a classification based on the type and density of immune cells inside the tumor, which can predict tumor prognosis more accurately than the traditional TNM system and guide the choice of immunotherapy for PCa (24). High-infiltration tumors with a high immune score are usually referred to as hot tumors, while low-infiltration tumors are referred to as cold tumors. BCR, a key intermediate event after RP, is suggestive of clinical recurrence or metastasis of PCa (34). Through this study, for the very first time, a recurrence risk signature at the polygenic level from the standpoint of MetS was constructed and validated to provide a reference for treatment decisions in clinical practice.

In this study, we first identified two MetS-related molecular clusters using consensus clustering. The two clusters had different tumor immune microenvironments. Unfortunately, there was no significant difference in BFS between the two clusters. Subsequently, we constructed the MSRPI using the LASSO multivariate Cox regression model. In the training cohort, test cohort, entire cohort, and validation cohort, the high-risk group was associated with poor BFS compared to the low-risk group. In the entire cohort, the area under the ROC curve for the MSRPI was 0.819 versus 0.773 for the Gleason score, demonstrating the outstanding predictive performance of this index for BFS. Multivariate Cox regression analysis revealed that the BFS-related MSRPI was an independent predictor of PCa. More importantly, the high- and low-risk groups exhibited distinctly different immune landscapes. The high-risk group featured more CD8+ and CD4+ T cell infiltration, higher immune scores, greater activation of immune checkpoints, and stronger sensitivity to immunotherapy, which are characteristic of hot tumors.

The MSRPI consists of seven metabolic syndrome-related genes (MSRGs), including *CSF3R*, *TMEM132A*, *STAB1*, *VIM*, *DUOXA1*, *PILRB*, and *SLC2A4*. Of these MSRGs, abnormal colony-stimulating factor receptor (CSFR) expression plays an oncogenic role in many hematologic and solid tumors, such as nasopharyngeal, breast, and ovarian cancers (35–37), but its role in PCa remains unclear. Transmembrane protein 132A (*TMEM132A*), a novel cellular pathway regulator, activates the Wnt pathway (38), the dysregulation of which underlies the metabolic traits of MetS (39). In addition, Wang et al. revealed that Wnt regulation drives prostate cancer bone metastasis and invasion (40). Stabilin-1 (*STAB1*) and triggering receptor expressed on myeloid cell 2 (*TREM2*) are expressed in a lipid-associated macrophage subset, which supports a tumor immunosuppressive microenvironment and is involved in the progression of obesity and its metabolic complications (41, 42). Cheaito et al. demonstrated that vimentin (*VIM*) could serve as a biomarker of epithelial-to-mesenchymal transition (EMT) to predict BFS in PCa (43). Ostrakhovitch et al. reported that cell proliferation inhibition associated with p21 upregulation occurs after transfection of the dual oxidase 1 (*DUOXA1*) and low expression of *DUOXA1* correlates with tumor aggressiveness (44). Moreover, the oxygen radicals produced by *DUOXA1* are associated with intravascular plaque formation, which is a critical risk factor for atherosclerosis (45). Using bioinformatics and immunohistochemistry approaches, Che et al. indicated that *PILRB* was enriched in high-risk PCa patients and could serve as a predictor of recurrence-free survival (46). Solute carrier family-2-member-4-gene (*SLC2A4*) is an insulin-sensitive glucose transporter protein that plays a key regulatory role in the pathogenesis of type 2 diabetes (47). Given that accelerated glycolysis initiated by glucose transport is essential for rapid proliferation in oncogenesis, it has been suggested that *SLC2A4* may serve as a prospective biomarker for a variety of tumors (48–50). Interestingly, although there have been few studies on the role of *SLC2A4* in PCa in recent years, Gonzalez-Menendez et al. revealed that *SLC2A4* appears to be more important for glucose uptake in androgen-insensitive PCa than in androgen-sensitive PCa (51).

We explored the somatic mutation landscape of patients with PCa in the TCGA cohort, where *TP53*, *TTN*, and *SPOP* were three of the high-frequency mutated genes. Mutations in *TP53*, a widely known and prominent tumor suppressor gene, can upregulate Twist1 expression and promote the epithelial-to-mesenchymal transition and recurrence in advanced PCa (52). Furthermore, mutant p53 proteins can aggregate, resulting in a negative effect on wild-type p53 proteins. Zhang et al. demonstrated the therapeutic potential of targeting mutant p53 proteins in PCa cells using a peptide inhibitor of p53 aggregation (53). There is a strong positive correlation between the number of Titin (*TTN*) mutations and TMB. Several studies have indicated that increased TMB correlates with improved response rates and survival benefits of immune checkpoint blockade therapy (54, 55). In our study, the high-risk group with more *TTN* mutations and higher TMB was associated with poor BFS and was more sensitive to immunotherapeutic agents, which was consistent with previous reports. Missense mutations in speckle-type POZ protein (*SPOP*) are one of the most common genetic mutations in PCa. Shi et al. showed that *SPOP* mutations prolonged the survival of PCa cells by upregulating cell cycle-associated protein 1 (Caprin1)-dependent stress granule assembly (56). It has also been reported that PCa-related *SPOP* mutants fail to ubiquitinate *SQSTM1*, promoting *SQSTM1*-dependent autophagy and exerting tumorigenic effects (57).

In this study, we constructed and externally validated a metabolic syndrome-associated risk model using seven genes. This risk formula contains fewer genes than similar studies, making its application simpler and easier. However, this study has some limitations. Firstly, we were not able to identify metabolic differences between the two clusters of PCa patients due to limitations of patient information in public databases. Basic experiments on the potential biological mechanisms of DEMSRGs are needed in the future. In addition, we need more real-world samples to verify the stability of the model.

## 5 Conclusion

In this study, 46 DEMSRGs were significantly associated with BFS, and two metabolic syndrome-associated molecular clusters were identified. The MSRPI is a potent and promising biochemical marker for PCa recurrence. Although PCa tends to have a barren tumor immune microenvironment, we were still able to categorize PCa tumors into hot and cold through MSRPI. Hot tumors appear to be more sensitive to immunotherapy and consequently, patients with hot tumors can benefit from this, providing a basis for personalized treatment options for PCa.

## Data availability statement

The original contributions presented in the study are included in the article/**Supplementary Material**. Further inquiries can be directed to the corresponding author.

## Author contributions

CR and XL designed the research. HL, YL and XC collected the data. CR, QW and SW performed the bioinformatics analysis. HZ and MX wrote the manuscript. All authors contributed to the article and approved the submitted version.

## Supplementary material

Supplementary Data Sheet 1 Differentially expressed genes in PCa from GEPIA2 database.

Supplementary Data Sheet 2 Metabolic syndrome-related gene network from MsigDB.

Supplementary Figure 1 Correlation test of 46 DEMSRGs with immune cell infiltration. *p < 0.05, **p < 0.01, ***p < 0.001, ****p < 0.0001.
