# Supplementary material for: Metabolic syndrome-related prognostic index: Predicting biochemical recurrence and differentiating between cold and hot tumors in prostate cancer
Source: Front Endocrinol (Lausanne). 2023 Mar 24;14:1148117. doi: 10.3389/fendo.2023.1148117 (PMC10080042; doi:10.3389/fendo.2023.1148117)
Supplement: Supplementary Data Sheet 1 — Differentially expressed genes in PCa from GEPIA2 database. [file DataSheet_1.docx]

| A1BG |
| --- |
| A1BG-AS1 |
| A4GALT |
| AASS |
| AATK |
| ABCA10 |
| ABCA6 |
| ABCA7 |
| ABCA8 |
| ABCA9 |
| ABCC10 |
| ABCC3 |
| ABCC4 |
| ABHD2 |
| ABO |
| ABTB1 |
| AC000089.3 |
| AC002116.8 |
| AC002398.12 |
| AC003090.1 |
| AC003973.3 |
| AC003991.3 |
| AC004540.5 |
| AC004854.4 |
| AC005042.4 |
| AC005104.3 |
| AC005154.6 |
| AC005154.7 |
| AC005253.2 |
| AC005255.3 |
| AC005336.5 |
| AC005517.3 |
| AC005519.4 |
| AC005538.3 |
| AC005943.2 |
| AC006128.2 |
| AC007009.1 |
| AC007326.1 |
| AC007566.10 |
| AC008746.12 |
| AC009065.4 |
| AC009120.6 |
| AC009133.21 |
| AC009948.5 |
| AC010761.9 |
| AC010976.2 |
| AC011290.5 |
| AC011558.5 |
| AC018462.3 |
| AC018766.4 |
| AC024560.3 |
| AC024937.6 |
| AC025165.8 |
| AC053503.12 |
| AC053503.6 |
| AC069213.1 |
| AC074289.1 |
| AC083843.1 |
| AC093110.3 |
| AC093495.4 |
| AC093642.3 |
| AC093838.4 |
| AC104667.3 |
| AC108488.4 |
| AC113188.2 |
| AC114730.11 |
| AC116366.6 |
| AC131097.4 |
| AC132217.4 |
| AC138035.2 |
| AC144450.1 |
| AC144530.1 |
| AC156455.1 |
| AC159540.1 |
| AC239868.2 |
| AC239868.3 |
| ACACA |
| ACACB |
| ACAD11 |
| ACADVL |
| ACAP3 |
| ACCS |
| ACHE |
| ACIN1 |
| ACKR3 |
| ACLY |
| ACOT11 |
| ACOX2 |
| ACSF2 |
| ACSL3 |
| ACSM1 |
| ACSM3 |
| ACSS3 |
| ACTA1 |
| ACTA2 |
| ACTA2-AS1 |
| ACTC1 |
| ACTG2 |
| ACVRL1 |
| ADA |
| ADAM10 |
| ADAM19 |
| ADAM28 |
| ADAM33 |
| ADAMTS1 |
| ADAMTS10 |
| ADAMTS2 |
| ADAMTS4 |
| ADAMTS8 |
| ADAMTS9 |
| ADAMTS9-AS1 |
| ADAMTS9-AS2 |
| ADAMTSL4 |
| ADAMTSL5 |
| ADAT2 |
| ADCY10P1 |
| ADCY3 |
| ADCY4 |
| ADCY5 |
| ADD3 |
| ADGRA2 |
| ADGRB1 |
| ADGRB2 |
| ADGRD1 |
| ADH1B |
| ADHFE1 |
| ADIRF-AS1 |
| ADM2 |
| ADM5 |
| ADPRHL1 |
| ADRA1A |
| ADRA1D |
| ADRA2C |
| ADRB1 |
| ADRB2 |
| AEBP1 |
| AF001548.3 |
| AF011889.2 |
| AF131217.1 |
| AFAP1L2 |
| AFG3L1P |
| AGA |
| AGAP11 |
| AGAP4 |
| AGAP5 |
| AGAP6 |
| AGAP9 |
| AGER |
| AGPAT4 |
| AGR2 |
| AGR3 |
| AHDC1 |
| AHI1 |
| AHNAK2 |
| AHSA2 |
| AIFM2 |
| AIM1 |
| AJUBA |
| AKAP12 |
| AKAP17A |
| AKAP8L |
| AKR1B1 |
| AKR1C1 |
| AKR1C2 |
| AL158801.1 |
| ALCAM |
| ALDH1A2 |
| ALDH1A3 |
| ALDH3B2 |
| ALDH6A1 |
| ALG14 |
| ALOX12P2 |
| ALPK1 |
| ALS2CL |
| AMACR |
| AMIGO2 |
| AMPD2 |
| AMT |
| AMY2B |
| ANAPC4 |
| ANGPT1 |
| ANGPTL1 |
| ANGPTL4 |
| ANK2 |
| ANKDD1A |
| ANKRD10 |
| ANKRD10-IT1 |
| ANKRD13D |
| ANKRD22 |
| ANKRD23 |
| ANKRD35 |
| ANKRD36 |
| ANKRD36B |
| ANKRD53 |
| ANKRD65 |
| ANKZF1 |
| ANO1 |
| ANO4 |
| ANO9 |
| ANXA2 |
| ANXA8 |
| AOC3 |
| AOX1 |
| AP000275.65 |
| AP000347.2 |
| AP000347.4 |
| AP000438.2 |
| AP000473.5 |
| AP000473.8 |
| AP000696.2 |
| AP000892.6 |
| AP001062.7 |
| AP001610.9 |
| AP003391.1 |
| AP006748.1 |
| AP1G2 |
| AP1M2 |
| AP1S1 |
| APBB1 |
| APBB3 |
| APLP2 |
| APOBEC3C |
| APOBEC3D |
| APOBEC3G |
| APOC1 |
| APOE |
| APOF |
| AQP1 |
| AQP11 |
| ARAP1-AS1 |
| ARAP3 |
| ARF6 |
| ARFGAP1 |
| ARFGEF3 |
| ARGLU1 |
| ARHGAP10 |
| ARHGAP23 |
| ARHGAP27 |
| ARHGAP33 |
| ARHGAP4 |
| ARHGAP40 |
| ARHGEF1 |
| ARHGEF10L |
| ARHGEF15 |
| ARHGEF17 |
| ARHGEF19 |
| ARHGEF2 |
| ARHGEF25 |
| ARHGEF26 |
| ARHGEF28 |
| ARHGEF4 |
| ARHGEF40 |
| ARID5A |
| ARL10 |
| ARL17A |
| ARL4D |
| ARL6IP1 |
| ARMCX1 |
| ARMCX4 |
| ARNTL |
| ARRDC2 |
| ASB2 |
| ASCL2 |
| ASIC3 |
| ASMTL-AS1 |
| ASPA |
| ASPHD1 |
| ASXL1 |
| ATAD3B |
| ATAD3C |
| ATG16L2 |
| ATG4B |
| ATHL1 |
| ATOH8 |
| ATP1A2 |
| ATP1B2 |
| ATP2A3 |
| ATP2B4 |
| ATP2C1 |
| ATP6AP1 |
| ATP6V0E2 |
| ATP6V1C2 |
| ATP6V1G1 |
| ATXN7L2 |
| AUTS2 |
| AVIL |
| AVPI1 |
| AXL |
| B3GAT1 |
| B3GNT8 |
| B3GNT9 |
| B4GALNT1 |
| BAMBI |
| BBS5 |
| BCAM |
| BCL11A |
| BCL6 |
| BCO2 |
| BDH2 |
| BDKRB2 |
| BEGAIN |
| BEND4 |
| BEND5 |
| BEST1 |
| BEX1 |
| BGLAP |
| BHLHA15 |
| BHMT2 |
| BIK |
| BIRC5 |
| BMP1 |
| BMP4 |
| BMP5 |
| BMPR1B |
| BMPR1B-AS1 |
| BNIPL |
| BOC |
| BOLA2B |
| BPIFB2 |
| BRI3BP |
| BRICD5 |
| BRSK1 |
| BTAF1 |
| BTBD19 |
| BTD |
| BTN2A1 |
| BTNL9 |
| BZRAP1 |
| C10orf54 |
| C10orf82 |
| C11orf96 |
| C12orf75 |
| C14orf159 |
| C15orf48 |
| C15orf52 |
| C15orf59 |
| C16orf45 |
| C16orf74 |
| C16orf89 |
| C19orf48 |
| C19orf66 |
| C1orf106 |
| C1orf116 |
| C1orf159 |
| C1orf162 |
| C1orf186 |
| C1orf233 |
| C1orf53 |
| C1orf95 |
| C1QL1 |
| C1QTNF1 |
| C1QTNF4 |
| C1RL-AS1 |
| C1S |
| C2 |
| C20orf166-AS1 |
| C20orf194 |
| C2orf40 |
| C2orf72 |
| C2orf88 |
| C3orf18 |
| C5orf30 |
| C5orf45 |
| C5orf56 |
| C6orf49 |
| C7 |
| C7orf55 |
| C8orf34-AS1 |
| C8orf46 |
| C8orf88 |
| C9orf152 |
| C9orf3 |
| CA14 |
| CA3 |
| CA4 |
| CABP1 |
| CABYR |
| CACHD1 |
| CACNA1C |
| CACNA1H |
| CACNB1 |
| CADM1 |
| CADM3-AS1 |
| CALHM2 |
| CALML6 |
| CALR |
| CAMK1G |
| CAMK2B |
| CAMK2G |
| CAMKK2 |
| CAND2 |
| CANT1 |
| CANX |
| CAPN10 |
| CAPN12 |
| CAPN3 |
| CAPN6 |
| CAPN8 |
| CAPRIN2 |
| CARTPT |
| CASP1 |
| CASQ1 |
| CATSPER2 |
| CAV1 |
| CAV2 |
| CBS |
| CBX7 |
| CCDC130 |
| CCDC136 |
| CCDC14 |
| CCDC146 |
| CCDC17 |
| CCDC178 |
| CCDC180 |
| CCDC3 |
| CCDC47 |
| CCDC66 |
| CCDC68 |
| CCDC69 |
| CCDC8 |
| CCDC80 |
| CCDC82 |
| CCDC84 |
| CCDC88A |
| CCDC93 |
| CCK |
| CCL14 |
| CCL18 |
| CCL23 |
| CCNB1 |
| CCND2 |
| CCND2-AS1 |
| CCNI2 |
| CCNL1 |
| CCNL2 |
| CCR10 |
| CD200 |
| CD24 |
| CD248 |
| CD40 |
| CDC20 |
| CDC25B |
| CDH1 |
| CDH19 |
| CDH23 |
| CDH24 |
| CDHR1 |
| CDK10 |
| CDK11A |
| CDK18 |
| CDK3 |
| CDKN1C |
| CDRT4 |
| CDS1 |
| CEACAM19 |
| CEBPB |
| CEBPD |
| CEL |
| CELF6 |
| CENPF |
| CENPJ |
| CENPM |
| CENPT |
| CEP164 |
| CEP95 |
| CERK |
| CERS4 |
| CES1 |
| CES4A |
| CFAP44 |
| CFAP70 |
| CFD |
| CFH |
| CFLAR |
| CGB7 |
| CGREF1 |
| CH17-258A22.4 |
| CH17-472G23.4 |
| CH507-42P11.8 |
| CH507-513H4.5 |
| CHIT1 |
| CHKB |
| CHMP4BP1 |
| CHMP4C |
| CHP2 |
| CHRD |
| CHRDL1 |
| CHST15 |
| CHST2 |
| CHST3 |
| CICP14 |
| CKM |
| CKS1BP3 |
| CLASRP |
| CLCN3 |
| CLCN6 |
| CLDN11 |
| CLDN15 |
| CLDN3 |
| CLDN4 |
| CLDN5 |
| CLDN7 |
| CLDN8 |
| CLEC1A |
| CLEC2B |
| CLEC2D |
| CLEC3B |
| CLGN |
| CLHC1 |
| CLIC6 |
| CLIP2 |
| CLIP3 |
| CLIP4 |
| CLK1 |
| CLK2 |
| CLK4 |
| CLMP |
| CLPS |
| CLU |
| CLUHP3 |
| CMAHP |
| CMTM4 |
| CMYA5 |
| CNN1 |
| CNN2 |
| CNRIP1 |
| CNTFR |
| CNTNAP1 |
| CNTNAP2 |
| CNTNAP3B |
| COL10A1 |
| COL13A1 |
| COL14A1 |
| COL16A1 |
| COL17A1 |
| COL18A1 |
| COL21A1 |
| COL23A1 |
| COL27A1 |
| COL4A2 |
| COL4A3 |
| COL4A4 |
| COL4A5 |
| COL4A6 |
| COL5A1 |
| COL5A2 |
| COL5A3 |
| COL6A1 |
| COL6A2 |
| COL6A3 |
| COL7A1 |
| COL9A1 |
| COL9A2 |
| COLCA2 |
| COLEC12 |
| COLQ |
| COMP |
| COPZ2 |
| COQ10A |
| CORO2A |
| CORO2B |
| CORO6 |
| CORO7 |
| COX4I2 |
| COX7CP1 |
| CPA6 |
| CPAMD8 |
| CPEB1 |
| CPLX1 |
| CPLX3 |
| CPNE5 |
| CPNE6 |
| CPT1C |
| CRABP2 |
| CRB3 |
| CREB3L1 |
| CREB3L4 |
| CRIP3 |
| CRIPAK |
| CRISP3 |
| CRISPLD2 |
| CRMP1 |
| CROCC |
| CROCCP3 |
| CRYAB |
| CRYGS |
| CSAD |
| CSDC2 |
| CSF1 |
| CSF3R |
| CSNK2B-LY6G5B-1181 |
| CSPG4 |
| CSPG4P10 |
| CSPG4P12 |
| CSRP1 |
| CSRP2 |
| CST2 |
| CSTA |
| CTA-14H9.5 |
| CTA-228A9.3 |
| CTB-13F3.1 |
| CTB-31O20.4 |
| CTB-50L17.8 |
| CTB-50L17.9 |
| CTB-60B18.18 |
| CTB-63M22.1 |
| CTB-79E8.3 |
| CTB-89H12.4 |
| CTC-251I16.1 |
| CTC-301O7.4 |
| CTC-429P9.4 |
| CTC-479C5.10 |
| CTC-510F12.2 |
| CTC-510F12.6 |
| CTC-524C5.2 |
| CTC-529I10.1 |
| CTC-529I10.2 |
| CTC1 |
| CTD-2012K14.6 |
| CTD-2013N17.6 |
| CTD-2013N24.2 |
| CTD-2020K17.1 |
| CTD-2020K17.4 |
| CTD-2026K11.6 |
| CTD-2033D15.2 |
| CTD-2036P10.6 |
| CTD-2054N24.2 |
| CTD-2089N3.1 |
| CTD-2192J16.22 |
| CTD-2196E14.6 |
| CTD-2224J9.4 |
| CTD-2228K2.7 |
| CTD-2270P14.1 |
| CTD-2311B13.5 |
| CTD-2314B22.1 |
| CTD-2314G24.2 |
| CTD-2373N4.3 |
| CTD-2396E7.11 |
| CTD-2516F10.2 |
| CTD-2517M22.14 |
| CTD-2527I21.14 |
| CTD-2537I9.12 |
| CTD-2537I9.18 |
| CTD-2545G14.4 |
| CTD-2547G23.4 |
| CTD-2547H18.1 |
| CTD-2595P9.4 |
| CTD-2649C14.2 |
| CTD-3035K23.7 |
| CTD-3092A11.2 |
| CTD-3128G10.6 |
| CTD-3141N22.1 |
| CTD-3157E16.2 |
| CTD-3193O13.11 |
| CTD-3252C9.4 |
| CTF1 |
| CTIF |
| CTNND2 |
| CTRB1 |
| CTTNBP2 |
| CUL9 |
| CWC25 |
| CXCL10 |
| CXCL11 |
| CXCL14 |
| CXCL17 |
| CXCL2 |
| CXCL9 |
| CXorf36 |
| CXXC1 |
| CYB5A |
| CYB5R2 |
| CYBA |
| CYGB |
| CYP27A1 |
| CYP2D6 |
| CYP2D7 |
| CYP2E1 |
| CYP2J2 |
| CYP39A1 |
| CYP3A5 |
| CYP4B1 |
| CYP4F12 |
| CYP4F22 |
| CYP4F29P |
| CYTL1 |
| DAAM2 |
| DAB2IP |
| DACT3 |
| DAPL1 |
| DBF4B |
| DBNDD1 |
| DBNDD2 |
| DCHS1 |
| DCN |
| DCST2 |
| DCUN1D4 |
| DDAH1 |
| DDB2 |
| DDIT4 |
| DDR2 |
| DDX11 |
| DDX17 |
| DDX26B |
| DDX3Y |
| DEFB1 |
| DEGS1 |
| DENND2A |
| DENND3 |
| DENND4B |
| DES |
| DFNB31 |
| DFNB59 |
| DGCR8 |
| DGKA |
| DGKH |
| DHCR24 |
| DHCR7 |
| DHDH |
| DHRS7 |
| DHX35 |
| DIO3OS |
| DKK3 |
| DLC1 |
| DLEC1 |
| DLG4 |
| DLGAP1-AS2 |
| DLK2 |
| DLL1 |
| DLX1 |
| DMD |
| DMKN |
| DMPK |
| DMTF1 |
| DNAH1 |
| DNAH5 |
| DNAJB5 |
| DNASE2B |
| DNHD1 |
| DNM1 |
| DNMBP-AS1 |
| DOC2A |
| DOCK6 |
| DOK4 |
| DONSON |
| DOPEY1 |
| DOPEY2 |
| DPH1 |
| DPH7 |
| DPP4 |
| DPT |
| DPY19L2 |
| DPYS |
| DPYSL3 |
| DRAIC |
| DSC2 |
| DSE |
| DSG2 |
| DSP |
| DST |
| DTNA |
| DTX3 |
| DTX4 |
| DUOX1 |
| DUOX2 |
| DUOXA1 |
| DUS3L |
| DUSP15 |
| DUX4L50 |
| DZIP1 |
| DZIP1L |
| EBF4 |
| ECHDC2 |
| ECHS1 |
| ECM1 |
| EDA2R |
| EDN3 |
| EDNRA |
| EDNRB |
| EEF1A1P12 |
| EEF1A1P5 |
| EEF1A1P6 |
| EEF1A1P9 |
| EEF2 |
| EFCAB1 |
| EFEMP1 |
| EFEMP2 |
| EFHC1 |
| EFHD1 |
| EFNB1 |
| EFS |
| EGFL7 |
| EGFL8 |
| EGFLAM |
| EHBP1L1 |
| EHD2 |
| EIF1AXP1 |
| EIF2AK1 |
| EIF4BP7 |
| EIF5AP4 |
| ELANE |
| ELF4 |
| ELFN1-AS1 |
| ELL3 |
| ELN |
| ELOVL2 |
| ELOVL7 |
| EMB |
| EME2 |
| EMILIN1 |
| EMILIN3 |
| EML3 |
| EMP3 |
| ENDOD1 |
| ENG |
| ENGASE |
| ENO2 |
| ENO3 |
| ENPP5 |
| ENPP7P11 |
| FGR |
| RAD52 |
| TMEM176A |
| TFPI |
| RBM5 |
| PLXND1 |
| RBM6 |
| HSPB6 |
| PDK4 |
| SPPL2B |
| ITGA3 |
| LAMP2 |
| OSBPL7 |
| MAP3K14 |
| TMEM132A |
| GGCT |
| GAS7 |
| MATK |
| RHBDF1 |
| LUC7L |
| TEAD3 |
| SPAG9 |
| ST3GAL1 |
| PKD1 |
| REV3L |
| MLXIPL |
| IFFO1 |
| GIPR |
| NISCH |
| STAB1 |
| FYN |
| MRC2 |
| NME2 |
| SEMA3B |
| KDM5D |
| MAMLD1 |
| MTMR11 |
| ISL1 |
| IGF1 |
| SLC38A5 |
| RUFY3 |
| PHLDB1 |
| PRDM11 |
| SNAI2 |
| SAMD4A |
| SERPINB1 |
| NRXN3 |
| FHL1 |
| PLEKHO1 |
| VIM |
| RTEL1-TNFRSF6B |
| MIPEP |
| HMGB3 |
| VCL |
| TIMP2 |
| USP2 |
| TUBG2 |
| FLT4 |
| PARP3 |
| FAM65C |
| HSPA5 |
| GPM6B |
| SCML1 |
| LMO3 |
| PRSS8 |
| NNAT |
| LAMA3 |
| KCNH2 |
| ITIH4 |
| TRAF1 |
| SOAT1 |
| PTPRU |
| HDAC7 |
| SLC6A16 |
| GPC1 |
| WISP2 |
| NGFR |
| SUGP2 |
| SLC12A2 |
| PDIA5 |
| MYLK |
| SEC61A2 |
| TLE2 |
| SLC9A7 |
| PDE4A |
| FGFR2 |
| STAG3 |
| TRO |
| RASGRP2 |
| PYGM |
| PITX1 |
| GAL |
| TGFBR3 |
| NUCB2 |
| FGF10 |
| FSTL3 |
| ST6GALNAC1 |
| GBA2 |
| ST6GALNAC2 |
| PABPC1 |
| OSBPL3 |
| SEL1L |
| TRIB2 |
| RDH11 |
| LIMS2 |
| SPEG |
| RHOBTB1 |
| MRVI1 |
| TP63 |
| PDE8A |
| GSDMB |
| FERMT2 |
| PTGS2 |
| GLI2 |
| SCARF1 |
| HACD3 |
| ZZEF1 |
| TUBE1 |
| WSCD2 |
| FOSL2 |
| SEC31B |
| RBMS2 |
| PLXNA2 |
| TPD52 |
| ICAM3 |
| MCAM |
| RAP1GAP |
| TM9SF3 |
| PPP1R12B |
| IL4R |
| NAALAD2 |
| FGFR1 |
| FBLN1 |
| ITGA8 |
| THOC1 |
| MKNK1 |
| TNS1 |
| RAPGEF3 |
| LIPE |
| PAFAH1B3 |
| EPB41L2 |
| SLC4A4 |
| KCNN2 |
| IGSF9B |
| SMARCD3 |
| PGR |
| EPB41L3 |
| TRAF5 |
| ZMPSTE24 |
| GSTP1 |
| NKAIN1 |
| MECOM |
| MGST2 |
| MAST2 |
| FOLH1 |
| FAT2 |
| TMED2 |
| TRIP6 |
| MMP2 |
| GNAO1 |
| NID2 |
| REM1 |
| TPX2 |
| FER1L4 |
| TMEM40 |
| SLC4A11 |
| ERP29 |
| LTBP4 |
| PCBP4 |
| IRAK3 |
| LYZ |
| FLT3LG |
| TNRC6A |
| PLEKHG2 |
| PITPNM2 |
| LAMB1 |
| SEL1L3 |
| TF |
| MYO15A |
| NLRP1 |
| JPH4 |
| SLC22A17 |
| TGM1 |
| UNC13D |
| XXbac-B461K10.4 |
| HDAC6 |
| FMO2 |
| EPB41L4B |
| TDRD1 |
| SORBS1 |
| SCD |
| HSD17B7P2 |
| PRTFDC1 |
| MZF1 |
| MYO9B |
| PRKY |
| LZTR1 |
| SEC14L2 |
| GGT1 |
| PRODH |
| TOP3B |
| LRP5L |
| SH3BP1 |
| LGALS1 |
| SOX10 |
| TTC28 |
| 3-Sep |
| TPTEP1 |
| KDELR3 |
| TIMP3 |
| SBF1 |
| RASD2 |
| SYNGR1 |
| FOXRED2 |
| KCTD17 |
| TRMU |
| HDAC10 |
| GNPNAT1 |
| GSTZ1 |
| RIN3 |
| PAPLN |
| REC8 |
| PNN |
| NFATC4 |
| MMP9 |
| NINL |
| SGK2 |
| MYBL2 |
| PABPC1L |
| SALL4 |
| SLCO4A1 |
| SLC17A9 |
| TRIB3 |
| SNPH |
| SEC23B |
| MYL9 |
| TLDC2 |
| WFDC2 |
| TNNC2 |
| ZNF516 |
| PAGE4 |
| SRPX |
| PLP2 |
| TAZ |
| SMS |
| GABRE |
| KLF8 |
| SRPX2 |
| SYTL4 |
| HSF4 |
| GDPD3 |
| TSNAXIP1 |
| PLLP |
| ZNF423 |
| NDRG4 |
| SLC7A6 |
| WDR59 |
| TAF1C |
| WFDC1 |
| FOXF1 |
| GGA2 |
| TOX3 |
| RRN3P2 |
| QPRT |
| MAZ |
| TMC5 |
| RASL12 |
| RAB11A |
| TMEM87A |
| SLC30A4 |
| EYA1 |
| IKBKB |
| WISP1 |
| GSDMD |
| TUSC3 |
| SNRNP70 |
| SYDE1 |
| NUMBL |
| TYK2 |
| RAB3D |
| RASIP1 |
| PDE4C |
| ISYNA1 |
| LSR |
| HPN |
| PBX4 |
| GRIK5 |
| RASA4 |
| PON3 |
| WDR91 |
| PTN |
| WNT2 |
| HOXA2 |
| LFNG |
| VIPR2 |
| STX1A |
| NOD1 |
| NSUN5P2 |
| NPTX2 |
| PTPRZ1 |
| PCOLCE |
| TSPAN13 |
| RARRES2 |
| OGN |
| KANK1 |
| NCS1 |
| GLIS3 |
| PTGDS |
| PDLIM1 |
| GATA3 |
| RASSF4 |
| UNC5B |
| SFXN3 |
| SH3PXD2A |
| MAP3K8 |
| KRT23 |
| RAPGEFL1 |
| RGS9 |
| P2RX1 |
| PIGL |
| SYNGR2 |
| KAT2A |
| EZH1 |
| SGCA |
| RND2 |
| LUC7L3 |
| MPP2 |
| HLF |
| FAM20A |
| YWHAE |
| MAP2K6 |
| WSB1 |
| MYH3 |
| TMEM97 |
| PMP22 |
| RAB34 |
| TMEM33 |
| GALNT7 |
| SLC2A9 |
| UGDH |
| PPARGC1A |
| FNBP4 |
| SC5D |
| VWA5A |
| TRIM3 |
| SLC35F2 |
| PITPNM1 |
| TCIRG1 |
| P3H3 |
| PRPF40B |
| KRT18 |
| TNS2 |
| GLI1 |
| PPM1H |
| TRPV4 |
| MYL2 |
| SCNN1A |
| OAS2 |
| MGP |
| STX2 |
| GNB3 |
| PHC1 |
| RP11-22B23.1 |
| FAM184A |
| FAM65B |
| FHL5 |
| EYA4 |
| SOBP |
| SLC39A7 |
| GMDS |
| VEGFA |
| LAMA4 |
| PCDHB2 |
| PAPD7 |
| THBS4 |
| RASGRF2 |
| LMNB1 |
| PPWD1 |
| WWC1 |
| TCERG1 |
| ERGIC1 |
| PDGFRB |
| NPHP3 |
| PCCB |
| RBP1 |
| HYAL1 |
| PODXL2 |
| PLSCR4 |
| ZBTB47 |
| TNNC1 |
| NKTR |
| KANSL3 |
| KCNIP3 |
| TFCP2L1 |
| POMC |
| LOXL3 |
| GALNT3 |
| REG1A |
| FN1 |
| IGFBP5 |
| OTX1 |
| ST3GAL5 |
| PLCD4 |
| WNT6 |
| IL1RL1 |
| IL18R1 |
| SLC9A2 |
| FHL2 |
| RND3 |
| EPAS1 |
| PRRX1 |
| QSOX1 |
| ERRFI1 |
| KIAA1324 |
| GON4L |
| FBXO2 |
| SRSF11 |
| ZBTB17 |
| RP11-268J15.5 |
| NID1 |
| KCNQ4 |
| UAP1 |
| GBP1 |
| ID3 |
| PRPF3 |
| TSPAN1 |
| HSD11B1 |
| RSRP1 |
| SLC35A3 |
| TNNT2 |
| NRP2 |
| USP35 |
| SGIP1 |
| PLAGL1 |
| SGK1 |
| TCF21 |
| PMFBP1 |
| RPN2 |
| RARRES1 |
| PHF19 |
| PGF |
| IFI27L2 |
| TGFB3 |
| SUPT7L |
| KLHL29 |
| EPCAM |
| SLC17A5 |
| PPP1R3C |
| KCNIP2 |
| SFRP5 |
| HOXB3 |
| LRP11 |
| MSANTD2 |
| SIL1 |
| MYOT |
| GLT8D2 |
| TNFRSF10B |
| LYPLA1 |
| RDH10 |
| TBX2 |
| TBX4 |
| TSHZ3 |
| KCNJ8 |
| POPDC2 |
| PILRB |
| FABP3 |
| LDB3 |
| ODF2L |
| NPY |
| POLM |
| TWIST1 |
| ZWINT |
| RBM19 |
| NECAB1 |
| PRDX4 |
| NLN |
| ITIH5 |
| MMP19 |
| NR4A1 |
| PDE1B |
| PLP1 |
| RAB9B |
| G0S2 |
| PLA2G12A |
| INHA |
| SLC12A4 |
| PLCG1 |
| TOX2 |
| PTGIS |
| STX16 |
| PPP4R1L |
| PMEPA1 |
| SEMG1 |
| HIF3A |
| PRICKLE4 |
| SPDEF |
| SOX4 |
| GLO1 |
| SSR1 |
| RAB17 |
| TMTC4 |
| TM9SF2 |
| PTGER2 |
| GGA3 |
| PPP1R12C |
| GRIA3 |
| TRIP10 |
| FOSB |
| TGM3 |
| FLRT3 |
| ID1 |
| KLC1 |
| XRCC3 |
| F10 |
| FFAR2 |
| NR1D1 |
| RRAS |
| GLIS2 |
| RHOJ |
| L3HYPDH |
| PLEKHG3 |
| TMEM35 |
| MASP1 |
| TSPAN8 |
| RAB3IP |
| SIN3B |
| SLC35E1 |
| KLF2 |
| PKMYT1 |
| GNG11 |
| SHFM1 |
| STEAP4 |
| PMS2P3 |
| SRD5A3 |
| PAICS |
| KDR |
| TUBGCP6 |
| POM121L9P |
| GNAZ |
| KRT17 |
| RNF112 |
| FLNC |
| HOXD9 |
| HOXD10 |
| HOXD11 |
| HOXD13 |
| PALLD |
| SOX15 |
| RIPK3 |
| FOXA1 |
| FGF13 |
| STARD8 |
| FAM155B |
| RPL36 |
| USHBP1 |
| STK33 |
| GDF15 |
| TRPM4 |
| TNNT3 |
| H19 |
| ZNF337 |
| GATA5 |
| LAMA5 |
| SMPDL3B |
| PNCK |
| HSD17B3 |
| RGN |
| SYNE1 |
| HIGD1B |
| GALNT15 |
| SLC6A6 |
| LRRC4B |
| PDLIM4 |
| GFPT2 |
| MAP1B |
| TNS4 |
| TOP2A |
| PPP1R1B |
| MCCC2 |
| RHPN2 |
| SPATA6 |
| SLC6A11 |
| NUP210 |
| TRIM22 |
| IQCA1 |
| SERPINF1 |
| PNISR |
| ITGB4 |
| TRIM47 |
| ZRANB2 |
| XAF1 |
| MATN2 |
| REEP2 |
| MTSS1L |
| SNAP25 |
| KIAA0907 |
| HAPLN2 |
| IGHMBP2 |
| MUTYH |
| LPIN3 |
| RBM38 |
| VSTM2L |
| LGR6 |
| SPG20 |
| TRPC4 |
| PRAM1 |
| SUV420H2 |
| MYH11 |
| GSTT2B |
| KRBA1 |
| ZNF767P |
| TMEM254 |
| LYVE1 |
| ZFC3H1 |
| MEIS2 |
| GSTM1 |
| GSTM5 |
| VAV3 |
| WNT2B |
| TRIM45 |
| LPIN1 |
| FST |
| NAV1 |
| ERN2 |
| RERG |
| LRP4 |
| FOLH1B |
| FHOD3 |
| SLC43A3 |
| FADS2 |
| PDGFRA |
| STT3A |
| GOLM1 |
| PSAT1 |
| NT5E |
| ITGA7 |
| RDH5 |
| PPP1R1A |
| FAIM2 |
| PAN2 |
| KRT7 |
| SLC26A10 |
| MAP7 |
| PKIB |
| MICAL1 |
| KIAA0513 |
| FHOD1 |
| KIAA1614 |
| TTLL4 |
| VILL |
| PHF11 |
| SPRY2 |
| LCP1 |
| KDELR2 |
| NACAD |
| MTHFS |
| SCN7A |
| GYPC |
| ST6GALNAC4 |
| IL11RA |
| SPAG8 |
| TJAP1 |
| TUBB2A |
| FOXF2 |
| IER3 |
| TLR2 |
| LRRC32 |
| MMP7 |
| TRIM29 |
| FXYD6 |
| MAPKBP1 |
| PARP6 |
| SMAD6 |
| SEMA6D |
| SPTBN5 |
| MYOF |
| PLCE1 |
| RBP4 |
| GPR87 |
| IDH1 |
| OLA1 |
| FAM117B |
| SHF |
| FGF2 |
| PDE5A |
| PRDM5 |
| NAAA |
| G3BP2 |
| PAPSS1 |
| PPP3CA |
| MAPK8IP3 |
| KIAA1644 |
| VAMP1 |
| PIANP |
| FAM222A |
| N4BP2L1 |
| MAP3K12 |
| LMBR1L |
| LPAR6 |
| NOVA1 |
| FRMD6 |
| RTN1 |
| FBLN5 |
| SLC27A2 |
| FGF7 |
| HDC |
| NEIL1 |
| MAN2C1 |
| TPM1 |
| PCSK6 |
| LMAN1L |
| NTRK3 |
| MFGE8 |
| TGFB1I1 |
| NLRC5 |
| KIFC3 |
| RHOT2 |
| MYOCD |
| KSR1 |
| SGSM2 |
| SLC39A6 |
| GALNT1 |
| SLC14A1 |
| MINK1 |
| SLC16A3 |
| RNF157 |
| RNF165 |
| PNMT |
| STAC2 |
| IGFBP4 |
| TMEM91 |
| SIK1 |
| SLC47A1 |
| KLK3 |
| RCN3 |
| SLC2A5 |
| EPHA2 |
| EVA1B |
| MAP3K6 |
| HSPG2 |
| TINAGL1 |
| PROK1 |
| ITGA10 |
| GPR161 |
| MPC2 |
| TBX19 |
| TMCO1 |
| PIGM |
| SEMA6C |
| POGZ |
| S100A8 |
| INTS3 |
| HCN3 |
| MBOAT2 |
| SOX13 |
| ETNK2 |
| OSR1 |
| PDIA6 |
| RHOB |
| MEIS1 |
| RABL2A |
| GPR17 |
| TMEFF2 |
| TRPM8 |
| STK11IP |
| RBMS3 |
| STAC |
| LRIG1 |
| NFKBIZ |
| PHLDB2 |
| PLA1A |
| SRPRB |
| TRPC1 |
| UCN2 |
| ILDR1 |
| MUC4 |
| SLIT2 |
| OCIAD2 |
| RNF175 |
| NKD2 |
| PLK2 |
| PIK3R1 |
| LHFPL2 |
| IQGAP2 |
| TSLP |
| KCNMB1 |
| KLHL3 |
| FAM193B |
| PLA2G7 |
| PPP1R18 |
| FGD2 |
| RNF217 |
| RSPO3 |
| SLC2A12 |
| SDK1 |
| SLC16A2 |
| OGT |
| ITGB1BP2 |
| ZNF711 |
| FBXO25 |
| ZNF185 |
| GNRH1 |
| MAL2 |
| STOM |
| GBGT1 |
| FAM73B |
| HMCN2 |
| INPP5E |
| NOTCH1 |
| ZEB1 |
| RGS10 |
| SCGB1A1 |
| SYT8 |
| PAMR1 |
| SERPING1 |
| SLC43A1 |
| NCAM1 |
| ST14 |
| PLCH2 |
| FEZ1 |
| ESAM |
| TAGLN |
| JPH2 |
| KIAA1755 |
| TM7SF2 |
| FAM124A |
| PRSS53 |
| NDUFC2 |
| ME3 |
| EPS8 |
| NCAPD3 |
| INPP1 |
| TMEM45B |
| SERP2 |
| SCHIP1 |
| HSPB8 |
| TCF7L1 |
| SPOCK1 |
| TRIM36 |
| ZFP36L2 |
| PLEKHH2 |
| SPARCL1 |
| GJA1 |
| PRDM8 |
| UTRN |
| MARVELD2 |
| TMEM87B |
| RBMS1 |
| SLC25A27 |
| FBXL2 |
| JAZF1 |
| PID1 |
| LGI4 |
| HS3ST3A1 |
| GDPD1 |
| ROBO4 |
| ROBO3 |
| PRKCA |
| UCHL1 |
| PGM5 |
| OBSCN |
| SH3RF1 |
| L3MBTL4 |
| PDE1C |
| SLFN13 |
| FLCN |
| PIEZO2 |
| PTPRN2 |
| MARVELD1 |
| SLC25A28 |
| PDIA4 |
| SLC26A2 |
| RAB39B |
| MICU3 |
| WIF1 |
| KCNMA1 |
| RBPMS |
| TIMP4 |
| NRG1 |
| STEAP2 |
| MMP14 |
| RP11-66N24.4 |
| KIT |
| TSC22D3 |
| ETS2 |
| TSPAN18 |
| WDR19 |
| PEX10 |
| MRPL17 |
| UBXN11 |
| HPD |
| TMSB15A |
| FAM46B |
| RNF207 |
| GPR153 |
| SLC13A3 |
| HIST1H4H |
| NRG2 |
| SLC45A3 |
| RNF166 |
| NBL1 |
| USF1 |
| ZNF276 |
| FGF17 |
| FAM160B2 |
| MPZ |
| PAXBP1 |
| SV2A |
| PRAC1 |
| HOXB13 |
| SIM2 |
| HK2 |
| STARD9 |
| ISL2 |
| SPON2 |
| ZYX |
| NPR2 |
| GNE |
| TFF3 |
| FTCD |
| S100B |
| ZNF208 |
| PPAPDC3 |
| TLCD1 |
| GBAP1 |
| NBEAL2 |
| PTH1R |
| MYL3 |
| FGFR4 |
| ZNF333 |
| SCGB3A1 |
| U2AF1L4 |
| PLXDC1 |
| ITGA5 |
| MPP3 |
| PLCD3 |
| GRASP |
| IP6K3 |
| WDR90 |
| NXF1 |
| TPCN2 |
| PPAP2B |
| SLC25A34 |
| PDPN |
| SCNN1D |
| MXRA8 |
| MEGF6 |
| NEXN |
| GBP2 |
| SNED1 |
| PKDCC |
| OXER1 |
| HAAO |
| PIGR |
| TRIM17 |
| SPATA18 |
| NOSTRIN |
| PDLIM5 |
| SPRR3 |
| S100A9 |
| TDRD10 |
| EOGT |
| LMOD1 |
| STK36 |
| FEV |
| TGFBR2 |
| NFASC |
| IFI16 |
| RPL22L1 |
| ICA1L |
| PRICKLE2 |
| SMIM14 |
| IL17RE |
| PCOLCE2 |
| TTC14 |
| TGM4 |
| ZC3H12A |
| RPN1 |
| S100P |
| PLXNB1 |
| INTU |
| MST1R |
| HAND2 |
| STARD4 |
| F2RL1 |
| SERINC5 |
| LEAP2 |
| PI16 |
| FBXL21 |
| STEAP1 |
| FABP5 |
| SBSPON |
| MICALL2 |
| OSR2 |
| GEM |
| FAM219A |
| MAMDC2 |
| FBP1 |
| TMEM246 |
| HECTD2 |
| ZCCHC24 |
| PHYHIPL |
| INPPL1 |
| VSTM4 |
| SLC18A2 |
| METTL17 |
| NDRG2 |
| NSMF |
| ZNF219 |
| HSPA12A |
| SLC39A13 |
| PDZRN4 |
| HACD1 |
| SMCO4 |
| GABRB3 |
| TMEM100 |
| MSS51 |
| TUB |
| RIC3 |
| ZMAT1 |
| ST5 |
| MFAP4 |
| MAPK7 |
| NDST2 |
| RIMKLB |
| TMED3 |
| SEC11C |
| TMEM135 |
| RRAD |
| MMP10 |
| TVP23A |
| PPIB |
| KIAA0101 |
| KIF7 |
| MESP1 |
| SCNN1G |
| RBPMS2 |
| NAV2 |
| GLYATL1 |
| NAB2 |
| STAT6 |
| NYAP1 |
| SMAD3 |
| MS4A8 |
| MAP1A |
| EVA1C |
| PDIA3 |
| NKX3-1 |
| SAMD14 |
| PIP5KL1 |
| PRRX2 |
| PRR15L |
| GPRC5B |
| TBC1D2B |
| IGF2 |
| RBFOX3 |
| ENTHD2 |
| OR51E2 |
| PRRT2 |
| SGK494 |
| NCKAP5L |
| NFKBID |
| LENG8 |
| PPP1R14A |
| SPINT2 |
| LY6D |
| PLIN4 |
| GPT |
| KIFC2 |
| SERPINF2 |
| GGT6 |
| KLK4 |
| ZNF83 |
| IGFBP6 |
| NUDT8 |
| TMEM88 |
| TK1 |
| SRRM2 |
| VWCE |
| RAB3IL1 |
| TRANK1 |
| TTC21A |
| LTBP3 |
| NAALADL1 |
| SCARA3 |
| SCARA5 |
| NT5DC2 |
| FAM107A |
| MLKL |
| SCNN1B |
| HR |
| RAB31 |
| TNXB |
| PHYHIP |
| GSTM4 |
| VAMP5 |
| INPP5D |
| JMJD7-PLA2G4B |
| MFSD7 |
| HNRNPH1 |
| ROR2 |
| NSMCE1 |
| RP11-231C14.4 |
| RAB3B |
| RSPO1 |
| THBS3 |
| NPIPB3 |
| KCNAB1 |
| SHE |
| NPR1 |
| ZEB2 |
| GJB1 |
| HEXDC |
| FASN |
| LDB2 |
| RAC3 |
| PCDH7 |
| REPS2 |
| NLGN2 |
| KCNAB3 |
| FAM153A |
| TMEM192 |
| HOXD4 |
| SLC16A5 |
| FAXDC2 |
| HSPB2 |
| FABP4 |
| TMED10 |
| KRT8 |
| KRT4 |
| SERPINB9 |
| SIX2 |
| STAT2 |
| RASA4B |
| TPT1-AS1 |
| PRKCDBP |
| LRRC8E |
| ZNF692 |
| NETO2 |
| ZNF439 |
| KRT15 |
| LURAP1 |
| KRT13 |
| PDE7B |
| MCC |
| PLEKHG5 |
| LGALS4 |
| GATM |
| ZNF540 |
| RRM2 |
| ZNF217 |
| SYNPO |
| MAL |
| LAMB2 |
| SLFN12 |
| MBOAT1 |
| ID4 |
| TPSAB1 |
| STARD5 |
| RCAN2 |
| SYNPO2 |
| GTPBP2 |
| RAB40A |
| SLFN11 |
| MUS81 |
| ZNF596 |
| NAA16 |
| RARG |
| NADSYN1 |
| MRGPRF |
| LCLAT1 |
| EVC2 |
| SNCG |
| FAM86B3P |
| STOX2 |
| TRIB1 |
| INSM1 |
| PPP1R14B |
| PEAK1 |
| TNFRSF10D |
| MST1 |
| LRFN4 |
| SLC19A1 |
| HSPB7 |
| PLK3 |
| GPR160 |
| SPTBN2 |
| HOXB2 |
| TCAP |
| FAM3C2 |
| MSRB3 |
| RP11-23P13.6 |
| PHLDA3 |
| GLIS1 |
| PODN |
| GTF2IRD2B |
| KLK15 |
| SLCO2A1 |
| ZNF266 |
| SH3PXD2B |
| RIN1 |
| SEZ6L2 |
| UBE2C |
| MARCKSL1 |
| PPM1E |
| NPPA |
| GOLGA8A |
| PHYHD1 |
| PHYKPL |
| KCNE3 |
| NR2F1 |
| PRIMA1 |
| ZNF169 |
| ETV4 |
| LRRN1 |
| GPX2 |
| LPCAT4 |
| SLCO3A1 |
| RNF152 |
| MUC20 |
| SCN4B |
| NUDT4P1 |
| ULK1 |
| HIC1 |
| PTRF |
| RPRM |
| PIDD1 |
| SRRM3 |
| NAALADL2 |
| PVRL3 |
| UBE2N |
| MAMDC4 |
| RNF212 |
| ZNF354B |
| GPR35 |
| STX19 |
| TRIM73 |
| LGALS7B |
| PER1 |
| FUCA1 |
| TMEM125 |
| MAGED1 |
| FAM156B |
| HMGN2P46 |
| PACS2 |
| LINC00174 |
| ZBTB42 |
| ZNF154 |
| TMEM86B |
| LYNX1 |
| HERC2P3 |
| GAS1 |
| HIST1H2BC |
| OR51E1 |
| LSMEM1 |
| MAPK15 |
| NPM1 |
| PENK |
| HIST3H2A |
| TMEM132C |
| SLX1B |
| SLC2A4 |
| TMEM259 |
| TMEM30B |
| RP11-69E11.4 |
| RGMA |
| UBA7 |
| FAM153B |
| SYNM |
| GABRG3 |
| SPACA6P |
| NXPH4 |
| WASH6P |
| FES |
| LINC01006 |
| TMEM198B |
| RRP7BP |
| RP11-181G12.2 |
| TCEAL7 |
| GJC1 |
| MTA1 |
| ZNF662 |
| PYCR1 |
| SPNS2 |
| PCP4 |
| SLC25A10 |
| LYSMD4 |
| GAS6 |
| PRR36 |
| PLGLB1 |
| EPHA10 |
| OVCH2 |
| NPIPA1 |
| RP11-958N24.1 |
| PI4KAP2 |
| FBXL7 |
| UPP1 |
| NPIPA5 |
| FAM162B |
| MAATS1 |
| FAM3B |
| KIRREL |
| SMTN |
| TMPRSS2 |
| FAM132A |
| GPR173 |
| TSPYL2 |
| GOLGA6L4 |
| POU6F1 |
| TACSTD2 |
| MROH7 |
| SRPK3 |
| SLIT3 |
| PLA2G6 |
| PABPC1L2B |
| KNTC1 |
| WDR27 |
| TMEM255B |
| TMEM173 |
| SMIM10 |
| TCTE3 |
| TMED9 |
| RBM33 |
| TCEAL2 |
| JAG2 |
| PTRHD1 |
| SORCS2 |
| FLRT2 |
| MANEAL |
| NRBP2 |
| IFITM2 |
| TNFAIP2 |
| SFTPA2 |
| SP140L |
| FAM174B |
| SDHAP1 |
| L3MBTL1 |
| OLFML2A |
| FAM212A |
| P4HB |
| NDUFA4L2 |
| ZFP36L1 |
| NTF3 |
| EP400NL |
| NPIPB4 |
| RNPC3 |
| TMLHE |
| KRT5 |
| SAPCD2 |
| 1-Mar |
| SOWAHB |
| MST1P2 |
| GLDN |
| KLK12 |
| MIR22HG |
| PDE2A |
| MST1L |
| TPCN1 |
| KRT16 |
| KRT14 |
| SERPINA11 |
| KANK3 |
| TMEM262 |
| ENTPD5 |
| SLIT1 |
| GCNT1 |
| MAGED4B |
| WDR86 |
| EPOR |
| LUZP2 |
| KCNJ11 |
| GJA4 |
| EXD3 |
| SAMD11 |
| PEAR1 |
| P2RX2 |
| PALM3 |
| LCN10 |
| LDLRAD2 |
| KLHL17 |
| NWD1 |
| NHS |
| SMTNL2 |
| LINC00265 |
| HNRNPU-AS1 |
| TUBB4B |
| PLA2G2A |
| SBK1 |
| FAM92A1 |
| ZP3 |
| IER5L |
| SERPINA5 |
| HBA2 |
| NPIPP1 |
| S100A16 |
| ZDHHC9 |
| PRELP |
| FBF1 |
| PLAC9 |
| UBE2Q2P1 |
| HN1 |
| ZNF600 |
| GJB5 |
| FAM150B |
| S100A14 |
| MMP23B |
| KIAA0895L |
| S100A4 |
| TMEM63A |
| MYO18A |
| LAMA2 |
| MYO6 |
| SDHAF3 |
| LINC00173 |
| PDXDC2P |
| ZNF700 |
| LAMB3 |
| PDLIM7 |
| FLNA |
| SMIM10L2B |
| ZNF300P1 |
| SLC25A29 |
| RP11-452G18.2 |
| SND1 |
| NEK5 |
| HIST1H4J |
| TPSB2 |
| KANK2 |
| SYNGAP1 |
| SVIL |
| ZNF655 |
| FBXL22 |
| MIB2 |
| OR51C1P |
| HOXC6 |
| KLHDC1 |
| OCLN |
| HIST1H2BK |
| SPG7 |
| FCHSD1 |
| S100A6 |
| GOLGA6L9 |
| SNHG12 |
| RP11-347C12.1 |
| SFI1 |
| LPAR1 |
| MB |
| ZNF251 |
| ZNF334 |
| SZT2 |
| RPS6KL1 |
| UCKL1 |
| HOXC4 |
| GFPT1 |
| NTRK1 |
| TPM2 |
| MAFK |
| ZNF789 |
| LRBA |
| MMP17 |
| PPIAP22 |
| MDM4 |
| FAM3D |
| SMOC1 |
| F5 |
| MT-ND2 |
| SELM |
| RUSC2 |
| GRK5 |
| SMC5 |
| MT-ATP6 |
| L1CAM |
| RPL39 |
| GPRASP1 |
| RNU4-1 |
| RN7SK |
| FAM83H-AS1 |
| SAMD5 |
| MSTO2P |
| SOX18 |
| LIME1 |
| SAMD13 |
| INPP5B |
| NPY4R |
| RXRB |
| HNRNPCP2 |
| NOTCH4 |
| PRRT1 |
| SMIM5 |
| LAYN |
| SLC44A4 |
| POU5F1 |
| RNF39 |
| HLA-J |
| FAM153C |
| GABBR1 |
| ZNF204P |
| ST8SIA6-AS1 |
| UQCC3 |
| ZNF783 |
| PRSS1 |
| TMEM240 |
| TRIQK |
| ZBTB10 |
| VIT |
| MUC12 |
| SAP25 |
| MT1A |
| EXOC3L4 |
| RGL3 |
| TMEM256 |
| RP11-1212A22.1 |
| RP9P |
| PPAPDC2 |
| RFPL3S |
| NYNRIN |
| HN1L |
| SERPINB11 |
| SERPINB5 |
| HERC2P9 |
| HBA1 |
| HACD2 |
| VGLL3 |
| PRSS50 |
| SNORA70 |
| GPX3 |
| IGLC1 |
| TRGC1 |
| TRGV10 |
| TRGV9 |
| IGHG4 |
| IGHG2 |
| IGHG1 |
| IGHG3 |
| LINC01089 |
| RPL15P3 |
| RP11-641D5.1 |
| RPS7P11 |
| MXD3 |
| GSTM2 |
| LCAT |
| RP11-75L1.2 |
| RPLP0P6 |
| SLX1A-SULT1A3 |
| LAT |
| S1PR3 |
| UBD |
| SLC23A3 |
| LTB4R |
| LTB4R2 |
| ITGA1 |
| ZNF90 |
| MEF2B |
| TTLL3 |
| TSPAN4 |
| PRCD |
| PLEKHM1P |
| SCART1 |
| RP11-252A24.2 |
| NEURL1B |
| LCNL1 |
| PLIN5 |
| RPL7P1 |
| MEG3 |
| HMGN2P15 |
| ZSWIM8 |
| SEPT7P2 |
| RP11-480I12.5 |
| HLA-F-AS1 |
| NEURL4 |
| RP11-266K4.9 |
| GOLGA8B |
| NPEPL1 |
| ZNF663P |
| RPL17-C18orf32 |
| HNRNPA1P7 |
| RP13-104F24.2 |
| TNFRSF25 |
| RPS2P55 |
| RP11-889L3.1 |
| FTH1P8 |
| RPL23AP47 |
| RP11-40C6.2 |
| ZGLP1 |
| MTMR9LP |
| SLC12A8 |
| FADS3 |
| TIAF1 |
| URAHP |
| RNY3P8 |
| MEG9 |
| RP11-632K20.7 |
| NSUN5P1 |
| RP4-717I23.3 |
| MIR503HG |
| TSSC2 |
| RP11-54O7.3 |
| FAM95B1 |
| RP11-80I15.1 |
| RP4-622L5.7 |
| RPS24P8 |
| UBE2SP2 |
| RP11-309L24.6 |
| MSL3P1 |
| SNRPGP15 |
| RPL29P19 |
| SH3BP5-AS1 |
| NPIPA3 |
| RP11-441O15.3 |
| RP11-82L18.2 |
| RP11-206L10.1 |
| PGM5-AS1 |
| INE1 |
| RP11-228B15.4 |
| XXbac-BPG308K3.5 |
| RP11-415J8.3 |
| RPL35P5 |
| MTND2P28 |
| SLC26A6 |
| FAM229A |
| RUSC1-AS1 |
| LINC00115 |
| RP11-384K6.2 |
| PCA3 |
| NTF4 |
| UBXN10-AS1 |
| KRT16P6 |
| RP11-419C5.2 |
| RP11-61N20.3 |
| RP11-157P1.4 |
| RP11-523H24.3 |
| RP5-1056L3.3 |
| FTH1P20 |
| RP11-128M1.1 |
| LENG8-AS1 |
| TBCAP1 |
| RP3-417G15.1 |
| RPS28P7 |
| TRGC2 |
| WASH7P |
| RP11-166N17.1 |
| RP4-669P10.19 |
| PCGEM1 |
| SPAG5-AS1 |
| RP11-488L18.4 |
| RP4-565E6.1 |
| MT-ATP8 |
| RP3-508I15.9 |
| GUSBP11 |
| LINC01004 |
| LINC01125 |
| RAB11FIP1P1 |
| SPCS2P4 |
| RP5-1160K1.6 |
| SAPCD1 |
| RP13-39P12.3 |
| VDAC1P8 |
| GS1-124K5.11 |
| PGA4 |
| HMGN1P37 |
| HMGN2P19 |
| RP11-274B21.12 |
| PRAC2 |
| PGA3 |
| RP11-399E6.4 |
| PRRT3-AS1 |
| HOXB-AS1 |
| RP3-508I15.19 |
| GOLGA6L5P |
| TCEA1P2 |
| MKX-AS1 |
| HCG4P7 |
| RP11-274B21.4 |
| LINC01001 |
| RPL10P3 |
| RP11-318C24.1 |
| RP3-525N10.2 |
| MIR205HG |
| RP4-631H13.6 |
| RP11-263K19.4 |
| RP1-207H1.3 |
| KLF3-AS1 |
| TRGVB |
| LINC01160 |
| LINC-PINT |
| PCAT7 |
| RP11-229P13.23 |
| IDH1-AS1 |
| PABPC1L2B-AS1 |
| RP11-311P8.2 |
| FTH1P7 |
| PRPS1P2 |
| GOLGA8N |
| LINC00342 |
| RP11-1212A22.4 |
| PHGR1 |
| RP11-295G20.2 |
| TMEM238 |
| TWIST2 |
| LINC01237 |
| YEATS2-AS1 |
| LINC01503 |
| RP11-250B2.3 |
| H2BFS |
| ZNF37BP |
| MAGI2-AS3 |
| PINLYP |
| RP11-166B2.1 |
| FOXP4-AS1 |
| SLC25A25-AS1 |
| RPL23AP42 |
| FABP5P7 |
| SEMA3F-AS1 |
| WI2-85898F10.1 |
| PPP1R3E |
| RP11-122K13.12 |
| FAM103A2P |
| SNRPGP10 |
| RP4-639F20.1 |
| RPS2P7 |
| H3F3AP4 |
| LINC00894 |
| HMGN1P36 |
| RP11-73M7.6 |
| LL0XNC01-240C2.1 |
| RP11-215A21.2 |
| HSPB1P1 |
| TMEM147-AS1 |
| TRHDE-AS1 |
| LINC00854 |
| RP11-175B9.3 |
| RPL13AP5 |
| POTEH-AS1 |
| LINC00106 |
| RP11-390F4.6 |
| RP4-669L17.10 |
| NR2F1-AS1 |
| SMG1P1 |
| TTN-AS1 |
| LINC01573 |
| LINC00844 |
| RPL35P1 |
| GOLGA2P5 |
| RP11-108M9.4 |
| SCARNA12 |
| RP11-464D20.2 |
| RPL23AP1 |
| RP11-295P9.3 |
| NME1 |
| RP4-800G7.1 |
| RN7SL608P |
| LY6G5B |
| SMKR1 |
| RP11-379F4.4 |
| RP11-499P20.2 |
| MTATP8P1 |
| RP5-890O3.9 |
| KRBOX1 |
| RP11-244H3.1 |
| RPL29P14 |
| RP11-147I3.1 |
| RP11-379B18.5 |
| RPL37P6 |
| IDS |
| PSMC1P1 |
| RP11-490G8.1 |
| INMT |
| RPL7AP30 |
| LINC00893 |
| LINC00969 |
| STAG3L5P |
| NPPA-AS1 |
| RP11-274B21.14 |
| MBL1P |
| RGAG4 |
| RP11-285F7.2 |
| RPL7P16 |
| MICAL3 |
| RP11-274B21.2 |
| MCCC1-AS1 |
| MNX1-AS1 |
| TNFRSF6B |
| RP11-379F4.1 |
| RP11-274B21.3 |
| RP5-966M1.6 |
| PLA2G4B |
| NPIPB5 |
| RPL23AP65 |
| RN7SL417P |
| RP11-148K1.12 |
| PKD1P1 |
| LY6G6D |
| RPL7P23 |
| SCARF2 |
| RP11-10G12.1 |
| LINC01213 |
| RP4-800G7.2 |
| FCGR2C |
| RP5-894A10.2 |
| HBB |
| RNF139-AS1 |
| NEAT1 |
| RAD51-AS1 |
| KB-1208A12.3 |
| RP11-696N14.1 |
| RP11-894P9.1 |
| RP11-1277A3.2 |
| H2AFJ |
| UBAP1L |
| MTND4P12 |
| RP11-1277A3.1 |
| NR2F2-AS1 |
| LINC00926 |
| FOXD1-AS1 |
| FAM13A-AS1 |
| RRN3P1 |
| WHAMMP2 |
| SEMA6A-AS1 |
| PCP4L1 |
| LINC00992 |
| LINC01207 |
| RP11-510N19.5 |
| ZNF436-AS1 |
| TMEM150C |
| RP11-496H1.1 |
| MIR143HG |
| YJEFN3 |
| FZD10-AS1 |
| PKD1P6 |
| RP11-356J5.12 |
| MGC32805 |
| GPR162 |
| RP4-669L17.8 |
| IQCJ-SCHIP1 |
| RP11-1084J3.3 |
| SNHG18 |
| RP11-849H4.4 |
| SHANK3 |
| RPL32P3 |
| FOXD1 |
| MALAT1 |
| RP11-582J16.5 |
| TMEM200B |
| RP1-170O19.17 |
| TRNP1 |
| PCAT1 |
| RP11-798K23.5 |
| HOXA-AS2 |
| IGHGP |
| KB-1562D12.1 |
| NPIPB11 |
| HSPB2-C11orf52 |
| RP11-755F10.3 |
| RP11-23F23.2 |
| RP5-1024C24.1 |
| RP11-481A20.10 |
| RP11-867G23.10 |
| FLJ20021 |
| RP11-395G23.3 |
| RP11-152H18.3 |
| RP11-468E2.1 |
| RP11-496I9.1 |
| RP11-867G23.1 |
| NAV2-AS1 |
| RP11-110I1.5 |
| RP11-326C3.7 |
| SCARNA9 |
| STX16-NPEPL1 |
| RP11-326C3.2 |
| RP11-802E16.3 |
| PIGY |
| MSH5-SAPCD1 |
| RP11-350N15.4 |
| RP11-142C4.6 |
| RP11-166D19.1 |
| RP11-732A19.5 |
| RP11-627G23.1 |
| RMST |
| RP11-197N18.2 |
| ZNF10 |
| SALL3 |
| RP11-73M18.2 |
| MRGPRF-AS1 |
| RP11-173P15.5 |
| KLRAP1 |
| ZNF350 |
| RP11-273B20.1 |
| SNRPEP2 |
| RP11-56G10.2 |
| RP11-278C7.1 |
| RP11-203J24.9 |
| PSMA3-AS1 |
| RP11-579D7.2 |
| RP3-416H24.1 |
| INAFM1 |
| RP11-478C19.2 |
| RP11-887P2.5 |
| RP11-644F5.10 |
| RTEL1 |
| RP11-649E7.5 |
| PPT2-EGFL8 |
| RP11-356O9.1 |
| LINC00641 |
| RP11-164J13.1 |
| RP11-1012A1.4 |
| RP11-192H23.4 |
| RP11-203M5.7 |
| RP11-66N24.3 |
| MC1R |
| HMGN1P3 |
| RP11-566K11.4 |
| RP11-589M4.1 |
| ITGB3 |
| RP11-182J1.12 |
| RP11-624L4.1 |
| RP11-109D20.2 |
| RP11-316M1.12 |
| RP11-279F6.2 |
| SORD2P |
| RP11-365N19.2 |
| RP11-89K11.1 |
| RP11-488L18.10 |
| RP11-304L19.1 |
| LA16c-390E6.4 |
| RP5-1142A6.9 |
| RP11-424G14.1 |
| RP11-483P21.2 |
| SNHG19 |
| RP11-44F21.5 |
| SLX1B-SULT1A4 |
| RP11-395I6.3 |
| RP11-645C24.5 |
| RP11-384K6.6 |
| RP11-63M22.2 |
| FAM157C |
| RP13-516M14.1 |
| RP11-20G6.3 |
| RP11-106M3.2 |
| RP11-72I8.1 |
| RP11-311C24.1 |
| LINC00890 |
| PRCAT47 |
| RP11-196G11.2 |
| RP11-96C23.11 |
| SULT1A3 |
| RP11-303E16.2 |
| RP11-264B17.3 |
| RP3-523K23.2 |
| RP11-304L19.3 |
| RP11-629O1.2 |
| LINC01355 |
| VPS9D1-AS1 |
| TEN1-CDK3 |
| RP4-616B8.4 |
| RP11-106M3.3 |
| UBE2MP1 |
| RP11-448G15.3 |
| LA16c-358B7.3 |
| SMG1P7 |
| RP11-414J4.2 |
| RP11-6O2.3 |
| RP11-554A11.4 |
| RP11-265N6.1 |
| RP11-401P9.4 |
| RP11-350O14.18 |
| MMP25-AS1 |
| RP11-473M20.9 |
| RP11-667K14.4 |
| SPON1 |
| RP11-1055B8.4 |
| RP11-235E17.6 |
| RP11-849I19.1 |
| MYZAP |
| RP1-59D14.5 |
| RP11-498C9.13 |
| RP11-927P21.5 |
| LINC00908 |
| RP11-159D12.2 |
| SUZ12P1 |
| RP11-186B7.4 |
| RP13-104F24.3 |
| PCAT18 |
| RP11-138I1.4 |
| RP11-524F11.1 |
| RP11-1376P16.2 |
| RP11-5A19.5 |
| RP11-452I5.2 |
| SNORA59B |
| RP11-159D12.5 |
| RASSF5 |
| RP5-890E16.4 |
| RP11-91I8.3 |
| MYO15B |
| SH3GL1P1 |
| RP13-104F24.1 |
| RP11-19P22.8 |
| FXYD1 |
| RP4-657D16.3 |
| RP11-677O4.6 |
| RP11-861E21.2 |
| LCN6 |
| RP11-793H13.10 |
| SNHG22 |
| UPK3BL |
| RP11-666A8.8 |
| ZNF224 |
| SMIM22 |
| RP11-167N5.5 |
| RP5-1057I20.4 |
| FAM156A |
| FENDRR |
| RP3-461F17.3 |
| RP11-34P13.15 |
| RP11-256I23.2 |
| TRABD2B |
| LINC01082 |
| FBXO17 |
| PTOV1-AS2 |
| RP3-461P17.10 |
| RP11-589N15.2 |
| RP11-932O9.9 |
| RP11-394O4.5 |
| RP11-73M18.7 |
| LUC7L2 |
| RP11-73M18.8 |
| RP5-940J5.9 |
| RP11-34P13.16 |
| LL0XNC01-7P3.1 |
| RP11-540B6.6 |
| MIR222HG |
| RP11-130L8.1 |
| MINOS1-NBL1 |
| RP11-258C19.7 |
| RP11-572O17.1 |
| RP11-373D23.3 |
| NBPF8 |
| RP11-373D23.2 |
| HSPE1-MOB4 |
| RP11-385F7.1 |
| RP11-346C20.3 |
| NUDT4P2 |
| RP11-171I2.4 |
| RP11-1109F11.5 |
| RP11-1109F11.3 |
| RP3-368A4.5 |
| RP3-368A4.6 |
| RP11-486G15.2 |
| SNX29P2 |
| MROH7-TTC4 |
| RP11-379F4.8 |
| RP1-244F24.1 |
| RP11-97C16.1 |
| RP4-635E18.8 |
| RP11-188P20.3 |
| RP11-285J16.1 |
| KB-431C1.5 |
| RP4-734G22.3 |
| RP11-155O18.6 |
| RP1-261G23.7 |
| RP11-465B22.8 |
| NFYC-AS1 |
| RP1-286D6.5 |
| RP11-802O23.3 |
| RP3-325F22.5 |
| HIST2H2AA4 |
| RP5-855D21.1 |
| RP11-346C20.4 |
| RP11-722E23.2 |
| RP3-329A5.8 |
| RP4-758J18.13 |
| RP5-1024N4.4 |
| MUSTN1 |
| ZSWIM8-AS1 |
| GS1-114I9.1 |
| RP11-504P24.8 |
| RP11-190A12.8 |
| RP3-508I15.21 |
| STAG3L5P-PVRIG2P-PILRB |
| RP11-326G21.1 |
| RP11-286H15.1 |
| RP11-260M2.1 |
| RP5-855D21.3 |
| RP3-402G11.27 |
| RP11-347I19.8 |
| RP11-17E13.2 |
| RP11-798M19.6 |
| RP11-408A13.4 |
| RP11-574K11.31 |
| RP11-500M8.7 |
| RP11-1191J2.5 |
| RP11-284F21.11 |
| KB-1572G7.2 |
| FAM95C |
| RP3-508I15.20 |
| PACERR |
| NBPF26 |
| RP3-402G11.28 |
| RP11-458F8.4 |
| RP4-583P15.15 |
| RP3-402G11.25 |
| RP11-527J8.1 |
| RP11-386I14.4 |
| RP5-1074L1.4 |
| RP13-514E23.2 |
| RP11-548H3.1 |
| RP11-390P24.1 |
| RP11-59C5.3 |
| RP11-7F17.8 |
| LL21NC02-21A1.1 |
| FAM27E3 |
| uc_338 |
| U1 |
| RP11-566K19.6 |
| RP11-44M6.7 |
| RP11-347I19.7 |
| PCA3_1 |
| RP11-128A17.2 |
| RP11-228M15.1 |
| TBC1D3L |
| PI4KAP1 |
| RP11-817I4.1 |
| RP11-395B7.2 |
| RP11-631N16.4 |
| LENG9 |
| RP13-516M14.10 |
| PRSS2 |
| RP11-680G24.6 |
| RP11-1055B8.9 |
| RP11-278C7.4 |
| TP53TG1_2 |
| RP11-481J2.4 |
| LLNLR-268E12.1 |
| HERC2P2 |
| RAB7B |
| RP5-1057I20.5 |
| RP1-102E24.10 |
| HOTAIRM1_2 |
| RP3-453C12.15 |
| RP11-102G14.1 |
| RMST_10 |
| RP11-837J7.4 |
| RP11-325L12.6 |
| RP11-147L13.13 |
| RP11-295M3.4 |
| HOTAIRM1_5 |
| RP11-734K23.9 |
| RP11-269C23.5 |
| SNORA76C |
| SRD5A2 |
| RP11-452N17.1 |
| RP11-736N17.10 |
| RP11-12A20.4 |
| RP11-467L19.16 |
| HIST1H2AM |
| NKILA |
| FAM27B |
| RP11-468E2.11 |
| HIST1H3H |
| RP11-640N20.4 |
| RP5-1039K5.12 |
| RP11-257O5.2 |
| RP11-574K11.24 |
| RP11-517I3.2 |
| LINC01451 |
| RP3-331H24.7 |
| RP11-449J21.3 |
| RP11-635L1.2 |
| RP11-369J21.12 |
| RPL41 |
| RP11-394B2.1 |
| RP11-574K11.32 |
| RP13-554M15.2 |
| LLNLF-158E9.1 |
| RP11-58O9.2 |
| RP11-1334A24.5 |
| RP11-43N16.4 |
| RP3-406P24.5 |
| LA16c-312E8.4 |
| RPL23AP61 |
| RP11-832A4.7 |
| RP11-87H9.4 |
| RP3-333H23.9 |
| MIR4697HG |
| RP11-1228E12.1 |
| RP11-972P1.7 |
| RP11-361L15.3 |
| PCAT14 |
| N4BP2L2-IT2 |
| U91328.1 |
| NPTN-IT1 |
| LINC01176 |
| RBM5-AS1 |
| WASH5P |
| LINC01002 |
