# Supplementary material for: Metabolic syndrome-related prognostic index: Predicting biochemical recurrence and differentiating between cold and hot tumors in prostate cancer
Source: Front Endocrinol (Lausanne). 2023 Mar 24;14:1148117. doi: 10.3389/fendo.2023.1148117 (PMC10080042; doi:10.3389/fendo.2023.1148117)
Supplement: Supplementary Data Sheet 2 — Metabolic syndrome-related gene network from MsigDB. [file DataSheet_2.docx]

| AAK1 |
| --- |
| ABCA5 |
| ABCB4 |
| ABCC4 |
| ABCC5 |
| ABCG1 |
| ABHD12 |
| ABI2 |
| ABI3 |
| ABL2 |
| ABR |
| ACE |
| ACER3 |
| ACOT9 |
| ACP2 |
| ACSS1 |
| ACTBL2 |
| ACTG1 |
| ACTL6B |
| ACTR3 |
| ACVRL1 |
| ACY1 |
| ADAM19 |
| ADAM8 |
| ADAMTS2 |
| ADAMTS3 |
| ADAP2 |
| ADCY6 |
| ADCY7 |
| ADGRE1 |
| ADGRE5 |
| ADPRH |
| ADSS1 |
| AFAP1L2 |
| AGMAT |
| AGPAT4 |
| AHCY |
| AIF1 |
| AIRN |
| AJAP1 |
| AJUBA |
| AK1 |
| AK8 |
| AKNA |
| AKR1A1 |
| AKR1B10 |
| ALDH18A1 |
| ALDH1B1 |
| ALDH3B1 |
| ALOX5AP |
| AMZ1 |
| ANAPC13 |
| ANG |
| ANKEF1 |
| ANKFY1 |
| ANKRD33B |
| ANO6 |
| ANPEP |
| ANTXR1 |
| ANXA2 |
| ANXA3 |
| ANXA4 |
| ANXA5 |
| AOPEP |
| AP2M1 |
| APBB1IP |
| APH1B |
| APOBEC1 |
| APOBEC3B |
| ARAP1 |
| ARHGAP10 |
| ARHGAP15 |
| ARHGAP18 |
| ARHGAP22 |
| ARHGAP25 |
| ARHGAP4 |
| ARHGAP45 |
| ARHGAP9 |
| ARHGDIA |
| ARHGDIB |
| ARID3A |
| ARL11 |
| ARL5C |
| ARMC2 |
| ARMC3 |
| ARMC7 |
| ARMCX2 |
| ARPC1B |
| ARPC2 |
| ARPC4 |
| ARPC5 |
| ARRB2 |
| ART4 |
| ASAH1 |
| ASF1B |
| ASGR1 |
| ASL |
| ATAD2 |
| ATF3 |
| ATP13A2 |
| ATP1A3 |
| ATP6AP2 |
| ATP6V0A1 |
| ATP6V0B |
| ATP6V0D2 |
| ATP6V1A |
| ATP6V1B2 |
| ATP6V1C1 |
| ATP8B2 |
| AUTS2 |
| AVPR1A |
| AZIN1 |
| B4GALT2 |
| B4GALT6 |
| B4GALT7 |
| BACH1 |
| BATF |
| BATF2 |
| BATF3 |
| BCL10 |
| BCL2A1 |
| BCL2L13 |
| BCS1L |
| BEX3 |
| BFAR |
| BGN |
| BICC1 |
| BIN3 |
| BIRC5 |
| BIRC7 |
| BLNK |
| BLVRA |
| BLVRB |
| BMP8B |
| BMPER |
| BST1 |
| BTC |
| BTG3 |
| C11orf86 |
| C16orf90 |
| C19orf38 |
| C19orf47 |
| C1QB |
| C1QC |
| C1orf100 |
| C1orf54 |
| C3AR1 |
| C3orf52 |
| C3orf80 |
| C4orf47 |
| C5 |
| C6orf62 |
| C8B |
| C8G |
| CA13 |
| CA2 |
| CACHD1 |
| CACNB3 |
| CADM1 |
| CADPS2 |
| CALHM2 |
| CALM3 |
| CALR |
| CAMKK1 |
| CAPG |
| CAPN2 |
| CAPNS2 |
| CARD11 |
| CARD19 |
| CASP1 |
| CASP12 |
| CASP2 |
| CAVIN1 |
| CBFB |
| CBR3 |
| CBX6 |
| CCDC102A |
| CCDC120 |
| CCDC148 |
| CCDC3 |
| CCDC80 |
| CCL15 |
| CCL23 |
| CCL3 |
| CCL4 |
| CCL7 |
| CCN4 |
| CCNA2 |
| CCR5 |
| CCRL2 |
| CD14 |
| CD160 |
| CD180 |
| CD200R1 |
| CD22 |
| CD244 |
| CD274 |
| CD276 |
| CD300A |
| CD34 |
| CD36 |
| CD37 |
| CD38 |
| CD3E |
| CD44 |
| CD48 |
| CD53 |
| CD5L |
| CD63 |
| CD68 |
| CD72 |
| CD74 |
| CD83 |
| CD84 |
| CD86 |
| CD9 |
| CDC42 |
| CDC7 |
| CDCA3 |
| CDCP2 |
| CDH3 |
| CDK15 |
| CDK18 |
| CDKN2C |
| CDS1 |
| CDT1 |
| CENPA |
| CERCAM |
| CERS5 |
| CERS6 |
| CES1 |
| CFAP45 |
| CFL1 |
| CFP |
| CH25H |
| CHCHD6 |
| CHCHD7 |
| CHST12 |
| CHST14 |
| CHSY1 |
| CHTF18 |
| CIDEA |
| CIDEC |
| CKLF |
| CLBA1 |
| CLCN5 |
| CLCNKB |
| CLDN22 |
| CLEC10A |
| CLEC1B |
| CLEC4A |
| CLEC4D |
| CLEC7A |
| CLIC1 |
| CLN8 |
| CLSPN |
| CMAS |
| CMTM3 |
| CNDP2 |
| CNMD |
| CNR2 |
| CNRIP1 |
| COL14A1 |
| COL16A1 |
| COL1A1 |
| COL1A2 |
| COL4A1 |
| COL5A1 |
| COL5A2 |
| COL6A1 |
| COL6A3 |
| COL8A1 |
| COLEC12 |
| COLGALT1 |
| COMMD10 |
| COMTD1 |
| CORO1A |
| COTL1 |
| CPNE9 |
| CPXM1 |
| CRAT |
| CREG1 |
| CRLF3 |
| CSF1R |
| CSF2RA |
| CSF2RB |
| CSF3R |
| CSTB |
| CTC1 |
| CTPS1 |
| CTSB |
| CTSD |
| CTSH |
| CTSK |
| CTSS |
| CTTNBP2NL |
| CXCL14 |
| CXCL16 |
| CXCL3 |
| CYBA |
| CYBB |
| CYBC1 |
| CYFIP1 |
| CYGB |
| CYP2C19 |
| CYP2D6 |
| CYP2E1 |
| CYP4F2 |
| CYP4F3 |
| CYP4F8 |
| CYRIB |
| CYSTM1 |
| CYTH4 |
| CYTIP |
| DAPP1 |
| DBH |
| DCDC2C |
| DCK |
| DCXR |
| DDAH2 |
| DDT |
| DDX31 |
| DENND1C |
| DENND4B |
| DGCR2 |
| DIAPH3 |
| DLG3 |
| DNA2 |
| DNAJB11 |
| DNASE1L1 |
| DNMT1 |
| DNMT3A |
| DOCK10 |
| DOCK11 |
| DOCK2 |
| DOCK8 |
| DOK2 |
| DOK3 |
| DPEP2 |
| DPP7 |
| DPYSL3 |
| DRAM1 |
| DROSHA |
| DSE |
| DUOXA1 |
| DUSP10 |
| DUSP18 |
| DUSP5 |
| DYNLL1 |
| DYSF |
| E2F8 |
| EAF1 |
| EBI3 |
| EBP |
| ECI2 |
| ECSCR |
| EFEMP2 |
| EGR2 |
| EHD2 |
| EHD4 |
| ELF3 |
| ELOVL1 |
| EMILIN1 |
| EML1 |
| EMP3 |
| ENAH |
| ENDOD1 |
| ENTPD1 |
| ENTPD2 |
| EPB41 |
| EPB41L2 |
| EPB41L3 |
| EPS8 |
| EPSTI1 |
| ERMP1 |
| ESPL1 |
| ETFB |
| EVA1A |
| EVI2A |
| EVL |
| EXOC3L4 |
| F10 |
| F11 |
| F13A1 |
| F2RL1 |
| F7 |
| FABP7 |
| FADS3 |
| FAM111A |
| FAM126A |
| FAM131A |
| FAM131B |
| FAM216A |
| FAM83A |
| FAM83F |
| FAR1 |
| FBLN2 |
| FBLN5 |
| FBLN7 |
| FBN1 |
| FBXO25 |
| FBXO32 |
| FCER1G |
| FCGR1A |
| FCGR2A |
| FCGR3A |
| FEN1 |
| FERMT3 |
| FES |
| FETUB |
| FGD6 |
| FHL1 |
| FHL2 |
| FILIP1L |
| FITM1 |
| FKBP10 |
| FKBP1A |
| FKBP1B |
| FLAD1 |
| FLNA |
| FLRT2 |
| FLT3 |
| FMC1 |
| FMN1 |
| FMNL3 |
| FMOD |
| FN3K |
| FOS |
| FOXP1 |
| FOXS1 |
| FRAT1 |
| FRMD4A |
| FRMD6 |
| FRRS1 |
| FRZB |
| FSCN1 |
| FSHR |
| FSTL4 |
| FUCA2 |
| FUT7 |
| FXYD5 |
| FZD5 |
| GADD45B |
| GAL3ST1 |
| GALNS |
| GALNT3 |
| GAS2L1 |
| GAS8 |
| GASK1B |
| GATA6 |
| GATM |
| GBA |
| GCH1 |
| GCNT1 |
| GDF10 |
| GDF3 |
| GDPD1 |
| GFRA4 |
| GGT5 |
| GIPC2 |
| GJB1 |
| GLB1 |
| GLIPR1 |
| GLRX |
| GMFG |
| GMIP |
| GMNN |
| GNB1 |
| GNB3 |
| GNE |
| GNG12 |
| GNMT |
| GNS |
| GPAT3 |
| GPATCH2 |
| GPATCH3 |
| GPLD1 |
| GPM6B |
| GPNMB |
| GPR137 |
| GPR137B |
| GPR179 |
| GPR65 |
| GPSM3 |
| GPX7 |
| GPX8 |
| GRN |
| GSAP |
| GSDMD |
| GSN |
| GSS |
| GSTA2 |
| GSTM1 |
| GUSB |
| HAAO |
| HACD4 |
| HAGH |
| HAL |
| HAUS8 |
| HAVCR2 |
| HBS1L |
| HCLS1 |
| HCST |
| HEATR1 |
| HEPH |
| HEXA |
| HEXB |
| HEY1 |
| HIC1 |
| HILPDA |
| HK2 |
| HK3 |
| HLA-DMB |
| HLA-DQA1 |
| HLA-G |
| HMCES |
| HMGA1 |
| HMOX2 |
| HOMER3 |
| HOXB2 |
| HOXD4 |
| HPCAL1 |
| HPGDS |
| HSD3B1 |
| HTR2B |
| HTRA1 |
| HTRA2 |
| HTRA3 |
| HVCN1 |
| ICA1 |
| ICOSLG |
| IER5 |
| IFI16 |
| IFI27 |
| IFI30 |
| IFIT2 |
| IFIT3 |
| IFNAR1 |
| IFNAR2 |
| IFNGR1 |
| IGFALS |
| IGFBP2 |
| IGSF6 |
| IGSF8 |
| IKBKE |
| IL10RA |
| IL10RB |
| IL11RA |
| IL17RD |
| IL18BP |
| IL1RL1 |
| IL33 |
| INIP |
| INPP5D |
| IQGAP2 |
| IRAG2 |
| IRF5 |
| IRF8 |
| ITGAD |
| ITGAL |
| ITGAX |
| ITGB2 |
| ITGBL1 |
| ITM2C |
| ITPR3 |
| ITPRID2 |
| JTB |
| JUN |
| KCNE3 |
| KCNK13 |
| KCNN2 |
| KCNN4 |
| KCTD10 |
| KCTD17 |
| KIF1C |
| KIF22 |
| KLC4 |
| KLF1 |
| KLHL6 |
| KLKB1 |
| KNG1 |
| KRT18 |
| KRT8 |
| KRTAP19-8 |
| KYAT1 |
| L1CAM |
| LACC1 |
| LACTB |
| LAIR1 |
| LAMA2 |
| LAMP2 |
| LAPTM5 |
| LASP1 |
| LAT2 |
| LAYN |
| LCE3B |
| LCP1 |
| LCP2 |
| LDHB |
| LDLRAP1 |
| LGALS1 |
| LGALS3 |
| LGALS3BP |
| LGI2 |
| LGMN |
| LGR4 |
| LHFPL6 |
| LILRA4 |
| LILRB3 |
| LILRB4 |
| LIPA |
| LIPC |
| LITAF |
| LMNA |
| LMO2 |
| LONRF3 |
| LOX |
| LOXL1 |
| LOXL2 |
| LPCAT2 |
| LPL |
| LPXN |
| LRCOL1 |
| LRIG1 |
| LRP12 |
| LRRC25 |
| LRRC27 |
| LRRC39 |
| LRRC52 |
| LRSAM1 |
| LTBP1 |
| LTBP2 |
| LTBP3 |
| LUM |
| LXN |
| LY86 |
| LYL1 |
| LYN |
| LYZ |
| MACIR |
| MAFB |
| MAGED2 |
| MAGIX |
| MAN1C1 |
| MAN2B1 |
| MANBA |
| MAP3K15 |
| MAP3K20 |
| MAP3K8 |
| MAP4 |
| MAPK7 |
| MATN2 |
| MCL1 |
| MCM3 |
| MCM5 |
| MCMBP |
| MCOLN2 |
| MCUB |
| MDFI |
| ME2 |
| MED11 |
| MEFV |
| MELTF |
| METRNL |
| MFAP2 |
| MFGE8 |
| MFHAS1 |
| MFRP |
| MICAL2 |
| MICU2 |
| MILR1 |
| MLANA |
| MMP12 |
| MMP13 |
| MMP19 |
| MMP2 |
| MMP23B |
| MMP27 |
| MMP3 |
| MOCOS |
| MOGAT1 |
| MPEG1 |
| MPND |
| MPV17L |
| MRC1 |
| MRE11 |
| MS4A6A |
| MS4A7 |
| MSANTD3 |
| MSC |
| MSR1 |
| MSX2 |
| MT3 |
| MTFP1 |
| MTHFD1 |
| MTHFD2 |
| MTHFD2L |
| MTHFS |
| MTMR11 |
| MTPN |
| MTRFR |
| MXRA8 |
| MYADM |
| MYL12A |
| MYL12B |
| MYO1E |
| MYO1F |
| MYO5A |
| MYO9B |
| MYOF |
| MYZAP |
| MZT2A |
| NAGK |
| NAIP |
| NANS |
| NAP1L1 |
| NAT8B |
| NCF2 |
| NCF4 |
| NCKAP1L |
| NDRG2 |
| NECAB1 |
| NEDD9 |
| NEURL2 |
| NFAM1 |
| NFE2L2 |
| NFKB2 |
| NIBAN1 |
| NIBAN2 |
| NID1 |
| NIN |
| NINJ1 |
| NIPAL3 |
| NLGN2 |
| NLRC3 |
| NLRC4 |
| NLRP10 |
| NLRP6 |
| NOL4L |
| NOSTRIN |
| NPC2 |
| NPDC1 |
| NPL |
| NQO1 |
| NRBP2 |
| NRG1 |
| NRP2 |
| NRROS |
| NUMBL |
| OACYLP |
| OAS1 |
| OASL |
| OASL2P |
| OBI1 |
| OBSL1 |
| OIT3 |
| OLFML3 |
| OLIG2 |
| OLR1 |
| OPN3 |
| OR4D6 |
| OR51M1 |
| OR56B4 |
| OR5P2 |
| OSBPL3 |
| OSBPL8 |
| OSTF1 |
| OTULINL |
| OXCT1 |
| P2RX4 |
| P2RY13 |
| P2RY14 |
| P2RY6 |
| P3H2 |
| P3H3 |
| PACC1 |
| PACSIN3 |
| PADI2 |
| PALB2 |
| PAM |
| PANX1 |
| PARP8 |
| PAWR |
| PBLD |
| PCDHB16 |
| PCDHB18P |
| PCGF3 |
| PCNA |
| PCOLCE |
| PDE3B |
| PDGFRB |
| PDGFRL |
| PDLIM4 |
| PEA15 |
| PECR |
| PEDS1 |
| PF4 |
| PFKP |
| PGM1 |
| PGS1 |
| PHF11 |
| PHLDB1 |
| PHYH |
| PI4K2A |
| PICALM |
| PIF1 |
| PIK3AP1 |
| PIK3IP1 |
| PILRA |
| PILRB |
| PIMREG |
| PIP4K2A |
| PIP4P2 |
| PIP5KL1 |
| PITPNM1 |
| PKIB |
| PKM |
| PKMYT1 |
| PLA2G4A |
| PLA2G7 |
| PLAC8 |
| PLAT |
| PLAUR |
| PLB1 |
| PLCD3 |
| PLCG2 |
| PLD3 |
| PLD4 |
| PLEK |
| PLEKHA4 |
| PLEKHB2 |
| PLEKHM2 |
| PLEKHN1 |
| PLEKHO1 |
| PLGRKT |
| PLIN4 |
| PLK2 |
| PLSCR1 |
| PLTP |
| PLXDC1 |
| PLXDC2 |
| PLXNB2 |
| PLXNB3 |
| PLXNC1 |
| PMAIP1 |
| PMP22 |
| POLD4 |
| POSTN |
| POU3F1 |
| PPARG |
| PPIB |
| PPIC |
| PPL |
| PPM1H |
| PPM1J |
| PPM1L |
| PPP1R14B |
| PRAF2 |
| PRCP |
| PRICKLE1 |
| PRKAG3 |
| PRKCD |
| PRKX |
| PRMT2 |
| PRODH |
| PRODH2 |
| PRR13 |
| PRR5L |
| PRRX2 |
| PRUNE1 |
| PSAP |
| PSD |
| PSMD8 |
| PSRC1 |
| PTCHD1 |
| PTGER2 |
| PTGIR |
| PTGR2 |
| PTK2B |
| PTPN1 |
| PTPN13 |
| PTPN18 |
| PTPN22 |
| PTPN6 |
| PTPRC |
| PTPRE |
| PTPRO |
| PXMP2 |
| PYCARD |
| RAB11FIP5 |
| RAB15 |
| RAB19 |
| RAB29 |
| RAB2A |
| RAB31 |
| RAB34 |
| RAB3IL1 |
| RAB7B |
| RAB8B |
| RAC2 |
| RALGDS |
| RAP2B |
| RASA1 |
| RASA3 |
| RASA4 |
| RASGEF1A |
| RASGEF1B |
| RASSF1 |
| RASSF4 |
| RASSF5 |
| RASSF8 |
| RBMS3 |
| RCN1 |
| RCN3 |
| RDH12 |
| REEP4 |
| RELB |
| RENBP |
| RFNG |
| RFTN2 |
| RGL1 |
| RGS1 |
| RGS10 |
| RGS14 |
| RGS18 |
| RGS19 |
| RGS2 |
| RHBDF1 |
| RHOC |
| RHOH |
| RHOJ |
| RHOV |
| RILPL2 |
| RIN2 |
| RINL |
| RIPK3 |
| RIPOR1 |
| RIPOR3 |
| RIPPLY3 |
| RNASE3 |
| RNASEH2B |
| RNF122 |
| RNF128 |
| RNF149 |
| RNF207 |
| RNF217 |
| RNH1 |
| RPE65 |
| RPIA |
| RPS6KA2 |
| RPS6KC1 |
| RTL6 |
| RUBCNL |
| S100A11 |
| S100A6 |
| S100A8 |
| S1PR2 |
| S1PR5 |
| SAMHD1 |
| SAMSN1 |
| SAT1 |
| SCCPDH |
| SCD |
| SCIMP |
| SCLY |
| SCN11A |
| SCNN1A |
| SCPEP1 |
| SDC2 |
| SDCBP2 |
| SDF2L1 |
| SDR42E1 |
| SEMA3B |
| SEMA3G |
| SEMA4D |
| SEMA5A |
| SEMA6D |
| SERPINA1 |
| SERPINA11 |
| SERPINA2 |
| SERPINB6 |
| SERPINB9 |
| SESN1 |
| SESTD1 |
| SGPL1 |
| SH3BGRL3 |
| SH3BP2 |
| SHANK1 |
| SHTN1 |
| SIGLEC5 |
| SIRPA |
| SKP2 |
| SLAMF6 |
| SLAMF7 |
| SLAMF8 |
| SLAMF9 |
| SLC11A1 |
| SLC11A2 |
| SLC15A3 |
| SLC1A5 |
| SLC22A7 |
| SLC25A10 |
| SLC25A23 |
| SLC25A24 |
| SLC25A36 |
| SLC27A3 |
| SLC27A5 |
| SLC27A6 |
| SLC2A10 |
| SLC2A4 |
| SLC2A6 |
| SLC31A1 |
| SLC35E4 |
| SLC35F6 |
| SLC37A2 |
| SLC39A12 |
| SLC39A6 |
| SLC41A2 |
| SLC43A1 |
| SLC48A1 |
| SLC4A11 |
| SLC4A3 |
| SLC4A7 |
| SLC5A4 |
| SLC66A2 |
| SLC6A6 |
| SLC6A8 |
| SLC7A6 |
| SLC7A8 |
| SLC8A1 |
| SLC9A9 |
| SLCO2A1 |
| SLCO2B1 |
| SLCO3A1 |
| SLK |
| SLPI |
| SMAP2 |
| SMC1B |
| SMIM5 |
| SMOC2 |
| SMOX |
| SMPDL3A |
| SMS |
| SNN |
| SNX1 |
| SNX20 |
| SNX24 |
| SNX27 |
| SNX5 |
| SOAT1 |
| SORT1 |
| SP100 |
| SPACA9 |
| SPARC |
| SPATA6 |
| SPATS2L |
| SPDL1 |
| SPEG |
| SPHK1 |
| SPI1 |
| SPIC |
| SPIN2A |
| SPOUT1 |
| SPRED1 |
| SPRED2 |
| SPRR1A |
| SPSB2 |
| SPTAN1 |
| SPTLC2 |
| SRD5A3 |
| SRGN |
| SRPX |
| SRPX2 |
| SRXN1 |
| SS18L2 |
| SSTR2 |
| ST6GALNAC4 |
| ST8SIA4 |
| STAB1 |
| STAMBPL1 |
| STARD3 |
| STAT4 |
| STEAP1 |
| STK10 |
| STK17B |
| STK24 |
| STRA6LP |
| STX4 |
| STX7 |
| STXBP1 |
| STXBP2 |
| STXBP3 |
| STXBP5 |
| SULF1 |
| SURF1 |
| SUSD3 |
| SUSD4 |
| SVEP1 |
| SYBU |
| SYK |
| SYNGR1 |
| SYNGR2 |
| SYNGR4 |
| SYNJ1 |
| SYNPO |
| SYP |
| TAGLN2 |
| TANC1 |
| TAOK3 |
| TAX1BP3 |
| TBC1D1 |
| TBC1D16 |
| TBC1D9 |
| TBPL1 |
| TCEAL1 |
| TCEAL5 |
| TEC |
| TEN1 |
| TEP1 |
| TFEC |
| TGFB1I1 |
| TGFB3 |
| TGFBR2 |
| THBS2 |
| THEMIS2 |
| TIFAB |
| TK1 |
| TLE4 |
| TLR2 |
| TLR4 |
| TLR7 |
| TM4SF4 |
| TM6SF1 |
| TMEM106A |
| TMEM119 |
| TMEM132A |
| TMEM144 |
| TMEM164 |
| TMEM165 |
| TMEM202 |
| TMEM229B |
| TMEM245 |
| TMEM273 |
| TMEM43 |
| TMEM51 |
| TMEM52B |
| TMEM86A |
| TMPRSS3 |
| TMSB4X |
| TNC |
| TNF |
| TNFAIP2 |
| TNFAIP6 |
| TNFAIP8 |
| TNFRSF18 |
| TNFSF13B |
| TNIP1 |
| TNIP2 |
| TNIP3 |
| TOM1 |
| TONSL |
| TOR2A |
| TOR4A |
| TPCN2 |
| TPD52 |
| TPH2 |
| TPM3 |
| TPST1 |
| TRAF5 |
| TRAM1L1 |
| TREH |
| TREM2 |
| TRERF1 |
| TREX1 |
| TRIL |
| TRIM32 |
| TRIM5 |
| TRIP13 |
| TRMT9B |
| TRPC4 |
| TRPV2 |
| TRUB2 |
| TSPAN33 |
| TSPAN6 |
| TTC28 |
| TTC39C |
| TUBA1A |
| TUBB |
| TUBB2A |
| TUBB4A |
| TUBB6 |
| TULP3 |
| TWF2 |
| TXNDC15 |
| TXNRD1 |
| TYRO3 |
| TYROBP |
| UBA7 |
| UBASH3B |
| UBD |
| UBTD1 |
| UBTD2 |
| UCK2 |
| UNC13D |
| UNC5B |
| UNC93B1 |
| UPB1 |
| USP12 |
| USP20 |
| USP43 |
| VAMP4 |
| VAMP8 |
| VASN |
| VAT1 |
| VAV1 |
| VCAM1 |
| VCAN |
| VIM |
| VIPAS39 |
| VOPP1 |
| VSIR |
| VTN |
| WAS |
| WASF1 |
| WDFY4 |
| WDR1 |
| WDR91 |
| WFDC3 |
| WFS1 |
| WLS |
| WSB2 |
| WT1 |
| YIPF7 |
| YWHAH |
| ZBTB48 |
| ZDHHC6 |
| ZFAND2A |
| ZFP90 |
| ZFYVE26 |
| ZNF385B |
| ZNF503 |
| ZNF521 |
| ZRANB3 |
