# Supplementary figures and images for: Metabolic syndrome-related prognostic index: Predicting biochemical recurrence and differentiating between cold and hot tumors in prostate cancer
Source: Front Endocrinol (Lausanne). 2023 Mar 24;14:1148117. doi: 10.3389/fendo.2023.1148117 (PMC10080042; doi:10.3389/fendo.2023.1148117)

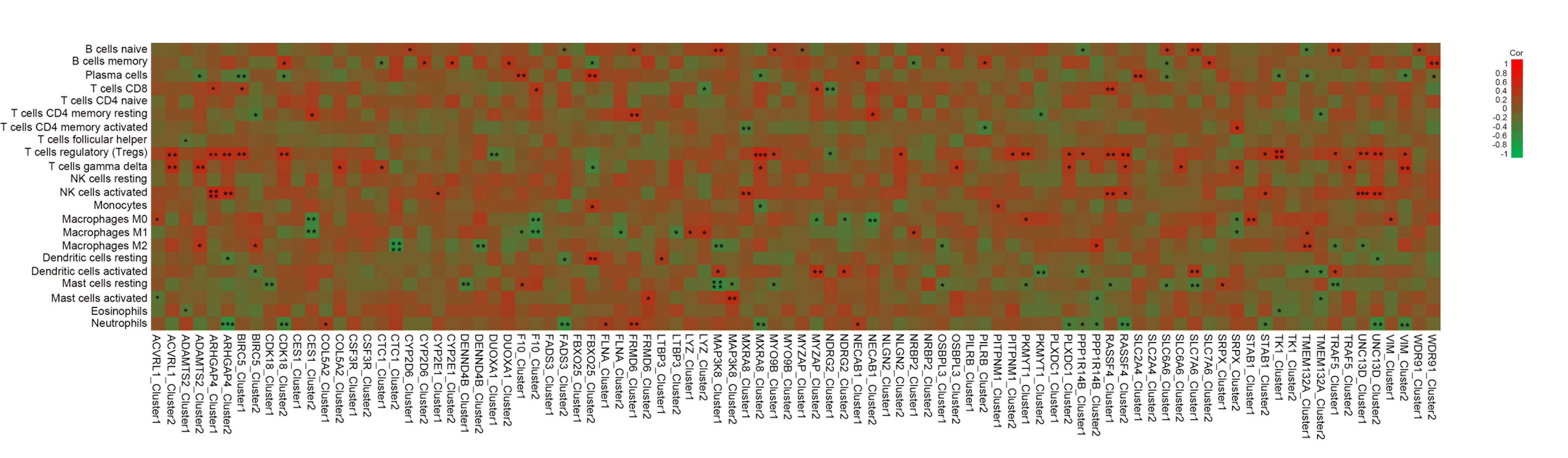

Supplement: Supplementary Figure 1 — Correlation test of 46 DEMSRGs with immune cell infiltration. *p < 0.05, **p < 0.01, ***p < 0.001, ****p < 0.0001. [file Image_1.tif]
